# Ascorbic acid as an adjunct therapy for anemia in erythropoietin-treated hemodialysis patients: a systematic review and meta-analysis

**DOI:** 10.1093/ckj/sfag070

**Published:** 2026-04-22

**Authors:** Salma Alejandra Beltrán Covarrubias, Angelo Magallanes Bajana, Alejandro Chen Liang, Yeison Cruz Castillo, Montserrat Villa Sánchez, España De la Rosa Valdez, Michelle Sámano Sánchez, Shreya Shambhavi, Vaidarshi Abbagoni, Oscar Yasser Pena Zapata, Camila Sanchez Cruz, José Antonio García-Erce, Ernesto Calderón-Martínez

**Affiliations:** Faculty of Medicine, Universidad de Guadalajara, Guadalajara, Mexico; Faculty of Medicine, Universidad De Guayaquil, Guayaquil, Ecuador; Faculty of Medicine, Centro de Estudios Universitarios Xochicalco Campus Tijuana, Tijuana, Mexico; Faculty of Medicine, Universidad Autónoma de Santo Domingo, Santo Domingo, Dominican Republic; Faculty of Medicine, Centro de Estudios Universitarios Xochicalco Campus Tijuana, Tijuana, Mexico; Faculty of Medicine, Universidad Autónoma de Nuevo León, Facultad de Medicina, Monterrey, Mexico; Faculty of Medicine, Universidad Autónoma de Sinaloa, Facultad de Medicina, Culiacán, Sinaloa, Mexico; Department of Internal Medicine, Rutgers Health/Community Medical Center, Toms River, NJ, USA; Department of Palliative and Hospice Care, Montefiore Medical Center, Albert Einstein School of Medicine Bonx, New York, USA; Department of Internal Medicine, St Vincent’s Medical Center / Quinnipiac University Frank H. Netter MD School of Medicine, Bridgeport, CT, USA; Faculty of Medicine, Universidad Nacional Autónoma de Mexico, Ciudad de México, Mexico; Banco de Sangre y Tejidos de Navarra, Servicio Navarro de Salud-Osasunbidea, Pamplona, Spain; Department of Internal Medicine, The University of Texas Health Science Center at Houston, Houston, Texas, USA

**Keywords:** chronic kidney disease, hemodialysis, hemoglobin, iron, vitamin C

## Abstract

**Background:**

Anemia is a common complication in chronic kidney disease (CKD), especially in hemodialysis patients, causing fatigue and reduced quality of life. Standard treatments involve iron and erythropoietin (EPO). Ascorbic acid (vitamin C) supports iron metabolism by converting ferric to ferrous iron and enhancing absorption and mobilization. This systematic review and meta-analysis evaluated the effectiveness of ascorbic acid in improving hematologic and iron parameters in adult anemic patients undergoing maintenance hemodialysis.

**Methods:**

Following Preferred Reporting Items for Systematic Reviews and Meta-Analyses (PRISMA) guidelines, a comprehensive search was performed in PubMed, EMBASE, Cochrane, Scopus, Web of Science, CINAHL and Google Scholar through May 2025. Only randomized controlled trials and crossover studies assessing ascorbic acid in adult anemic hemodialysis patients were included. The protocol was registered in the International Prospective Register of Systematic Review (PROSPERO) (CRD420251056337). Primary outcomes were hemoglobin (Hb), ferritin, serum iron, transferrin saturation (TSAT) and total iron-binding capacity (TIBC). Meta-analyses used DerSimonian-Laird random-effects models.

**Results:**

Of 479 screened articles, 14 studies were included. Ascorbic acid supplementation was associated with a modest but significant increase in Hb [mean difference (MD) 0.94 g/dL; 95% confidence interval (CI) 0.57 to 1.31; *P* < .01; *I*² = 87.2%] and TSAT (MD 6.86%; 95% CI 1.93 to 11.78; *P* < .01; *I*² = 96.7%). Ferritin levels showed a slight but significant reduction (MD −65.00 ng/mL; 95% CI −117.20 to −12.80; *P* = .01; *I*² = 57.2%), along with a decrease in TIBC (MD −22.54 µg/dL; 95% CI −42.37 to −2.72; *P* = .03; *I*² = 76.1%). EPO requirements expressed as units/kg/week were significantly reduced (MD −21.29; 95% CI −27.73 to −14.84; *P* < .01; *I*² = 0.0%). No significant changes were observed in serum iron levels or EPO dosage expressed as IU/week.

**Conclusion:**

Ascorbic acid supplementation may confer modest hematologic benefits in hemodialysis patients with improvements in Hb, TSAT and iron utilization, and a small reduction in weight-adjusted erythropoiesis-stimulating agent dose. However, the certainty of evidence is low, and long-term safety and patient-centered outcomes remain unestablished.

## INTRODUCTION

The World Health Organization (WHO) classically defines anemia in adults as a hemoglobin (Hb) level <12 g/dL for women and <13 g/dL for men [1]. Anemia is estimated to be present in up to 95% of patients undergoing hemodialysis, and its prevalence increases as estimated glomerular filtration rate declines [2, 3]. This multifactorial condition results from relative erythropoietin (EPO) deficiency, accumulation of uremic inhibitors of erythropoiesis, shortened erythrocyte lifespan, disordered iron homeostasis, and blood losses related to phlebotomy, anticoagulation, uremic thrombocytopathy and the dialysis procedure [4]. Anemia in chronic kidney disease (CKD) imposes a significant clinical burden, presenting with fatigue, dyspnea, headaches, palpitations, lightheadedness and a general decline in health-related quality of life [5]. Moreover, it has been associated with an increased risk for cardiovascular disease and all-cause mortality, as demonstrated in prior studies. In addition to its clinical implications, anemia in CKD represents a considerable economic burden on healthcare systems [6].

The Kidney Disease: Improving Global Outcomes (KDIGO) guidelines currently recommend erythropoiesis-stimulating agents (ESAs) combined with iron supplementation as the gold standard for CKD-related anemia [7]. According to the US Renal Data System 2020 Annual Data Report, >85% of hemodialysis patients receive ESAs [8]. Although clinical practice guidelines recommend the use of ESAs as the gold standard to address anemia, a subset of patients exhibit resistance or inadequate responsiveness. This is largely due to the multifactorial nature of anemia in this population. Adjuvant therapies, such as zinc, ferric citrate, vitamin C and vitamin E, have been explored to enhance ESA responsiveness [9]; nonetheless, current guidelines do not provide clear recommendations regarding their use [9].

Vitamin C plays a key biochemical role in iron metabolism by catalyzing the reduction of ferric (Fe^3+^) to ferrous (Fe^2+^) iron, the form required for intestinal absorption and erythropoiesis [10]. This reduction step is essential for non-heme iron uptake: Fe^3+^ is converted to Fe^2+^ by duodenal cytochrome b and then transported via divalent metal transporter 1 (DMT1). Vitamin C enhances this process within the gastrointestinal lumen, with studies showing that co-ingestion of 500 mg of vitamin C with a meal increases iron absorption 6-fold, while the same dose taken separately has no effect, highlighting the importance of luminal Fe^3+^-to-Fe^2+^ conversion at the time of iron exposure [11].

Moreover, insufficient ascorbic acid intake has been associated with increased microvascular fragility and gingival bleeding [12]. In patients with upper gastrointestinal bleeding, low ascorbic acid levels have been linked to adverse clinical outcomes, including higher mortality, increased risk of rebleeding, and prolonged hospital stay [13]. Despite evidence indicating that plasma vitamin C levels are often low in dialyzed patients [11], this recommendation has not been incorporated into standard anemia management protocols.

While the previous abstract has examined the use of vitamin C in this patient population, there is still a need for updated evidence from studies with larger databases to effectively evaluate its efficacy in reducing inflammation, thus improving Hb levels and decreasing the occurrence of complications. This systematic review and meta-analysis address this critical gap, synthesizing available evidence to assess the impact of ascorbic acid supplementation as an adjunct to EPO therapy in adult hemodialysis patients with anemia. The goal is to inform clinical decision-making and guide future research evaluating outcomes such as Hb (g/dL), serum ferritin (ng/mL), serum iron (µg/dL), total iron-binding capacity (TIBC; µg/dL), transferrin saturation (TSAT; %), EPO dose (IU/week) and C-reactive protein (CRP; mg/L).

## MATERIALS AND METHODS

The present systematic review followed the recommendations and criteria established by the Preferred Reporting Items for Systematic Reviews and Meta-analyses (PRISMA) [14] reporting guidelines. The protocol was pre-registered at the International Prospective Register of Systematic Review (PROSPERO) [15] with the identifier code CRD420251056337.

### Eligibility criteria

#### Types of study

This review includes studies published from the inception of the database to 17 May 2025. Eligible study designs include randomized controlled trials and crossover studies. Additionally, studies were excluded if they lack sufficient methodological detail, present duplicate data or fail to provide the required information after attempts to contact the original authors.

#### Types of participants

The review includes participants diagnosed with anemia and undergoing dialysis. The inclusion criteria consisted of an adult population defined as patients >18 years, with formal diagnosis of anemia and undergoing any type of dialysis. Exclusion criteria include individuals <18 years, patients not undergoing dialysis or patients without anemia.

#### Types of interventions/exposures and comparators

This review evaluates the effects of vitamin C (ascorbic acid) on the study population, administered either orally or intravenously (IV). The reported doses ranged from 1500 mg to 7500 mg, with administration frequencies varying from 3 to 30 times per month. Comparators include patients receiving placebo or regular treatment, involving iron and EPO administered IV. Exclusion criteria will apply to interventions or exposures that are not clearly defined or reported in the study.

### Outcomes

The primary outcomes of this review were Hb (g/dL), serum ferritin (ng/mL), serum iron (µg/dL), TSAT (%),TIBC (µg/dL) and ESA dose (IU/week or IU/kg/week). Secondary outcomes included CRP (mg/L). Studies were excluded if they did not report data for at least one predefined outcome.

### Searching methods

A systematic search was initially conducted on 17 May 2025, on PubMed/MEDLINE, Cochrane, Scopus, Web of Science, EMBASE, CINAHL and Google Scholar using the following terms: Anemia, Hemodialysis, Chronic Kidney Disease, Erythropoietin, and adults. All detailed search strategies can be found in the Supplementary data, Tables S1–S7.

### Selection of studies

All references were exported to Rayyan (Rayyan Systems Inc., Cambridge, MA, USA), and duplicates were removed [16]. Two authors independently (A.C.L. and M.V.S.) completed the eligibility assessment, first by title and abstract analysis and, subsequently, by full-text assessment. In disagreements between reviewers, a third reviewer (Y.C.C.) helped reach a consensus.

### Data extraction

Two independent reviewers extracted the data (E.C.-M. and Y.C.C.), and disagreements were resolved by consensus. When multiple overlapping reports from the same study were identified, the information from the one containing the most relevant information or the first published report was included. Extracted data included sample sizes, intervention types and measured outcomes. Conventional methods were used for data extraction, complemented with specialized tools such as WebPlotDigitizer (Automeris, Austin, TX, USA) [17] for digitizing data from graphs, Cochrane Calculator for statistical conversions [18] and StatsToDo for advanced calculations [19]. Change from baseline was calculated as the difference between baseline and final values. These outcomes compromised Hb (g/dL), serum ferritin (ng/mL), serum iron (µg/dL), EPO dose (IU/week), TSAT (%), TIBC (µg/dL), ESA dose and CRP (mg/L). ESA dose was extracted as reported and categorized according to unit of measurement (IU/week or IU/kg/week). When both metrics were available, IU/kg/week was prioritized for analysis due to improved clinical comparability. Dialysis duration was intended for extraction, however none of trials included reported this information. Additional extracted data included subgroup characteristics, such as country of study, risk of bias levels and study design.

### Assessment of risk of bias in included studies

To assess the quality of the studies included in the systematic review, according to the Cochrane guidelines we applied the Cochrane RoB 2.0 tool [20] for randomized controlled trials (RCTs). Two independent reviewers (A.C.L. and M.V.S.) evaluated the risk of bias in each study, considering the specific criteria and guidelines provided by the respective tools. Any reviewer discrepancies were resolved through discussion with a third, blinded reviewer (S.A.B.C.).

### Assessment of the certainty of the evidence and summary of findings

Two reviewers independently (A.C.L. and Y.C.C.) assessed the overall quality of evidence for each outcome using the five domains of the Grading of Recommendations Assessment, Development, and Evaluation (GRADE) [21] approach, study design, risk of bias, inconsistency, indirectness, imprecision, publication bias, magnitude of effect, cofounding and dose–response gradient, via the GRADEpro GDT software [22]. No discrepancy was observed between reviewers.

### Statistical analysis

A meta-analysis was performed using R version 3.4.3 (R Core Team) with the meta and metafor packages. The pooled effect of the outcomes was examined using a random-effects meta-analysis (DerSimonian-Laird approach). Whenever the number of studies reporting an outcome of interest was insufficient, only a qualitative analysis of the results was performed. Effect sizes were expressed as mean difference (MD) with a 95% confidence interval (CI). The *I*² statistic assessed heterogeneity, and the following cut-off values were used for interpretation: <25%, 25%–50% and >50% were considered small, medium and large heterogeneity, respectively. For crossover trials, only data from the first treatment period were included in the meta-analysis to minimize potential carryover effects, in accordance with recommended methodological guidance for crossover study synthesis [23]. For all outcomes, sensitivity analyses using the leave-one-out method were performed to determine the influence of individual studies on the overall effect. Egger’s regression test was used to examine publication bias when 10 or more reports with the same outcome were available. Whenever possible, subgroup analyses were planned based on risk of bias and performed in primary outcomes.

### Ethics statement and consent for publication

Given that this meta-analysis utilized previously published data from studies that had already obtained ethics approval and consent to participate, no additional ethics approval or consent was required for this research. All authors have provided their consent for publication.

## RESULTS

### Study selection

**Figure 1:**
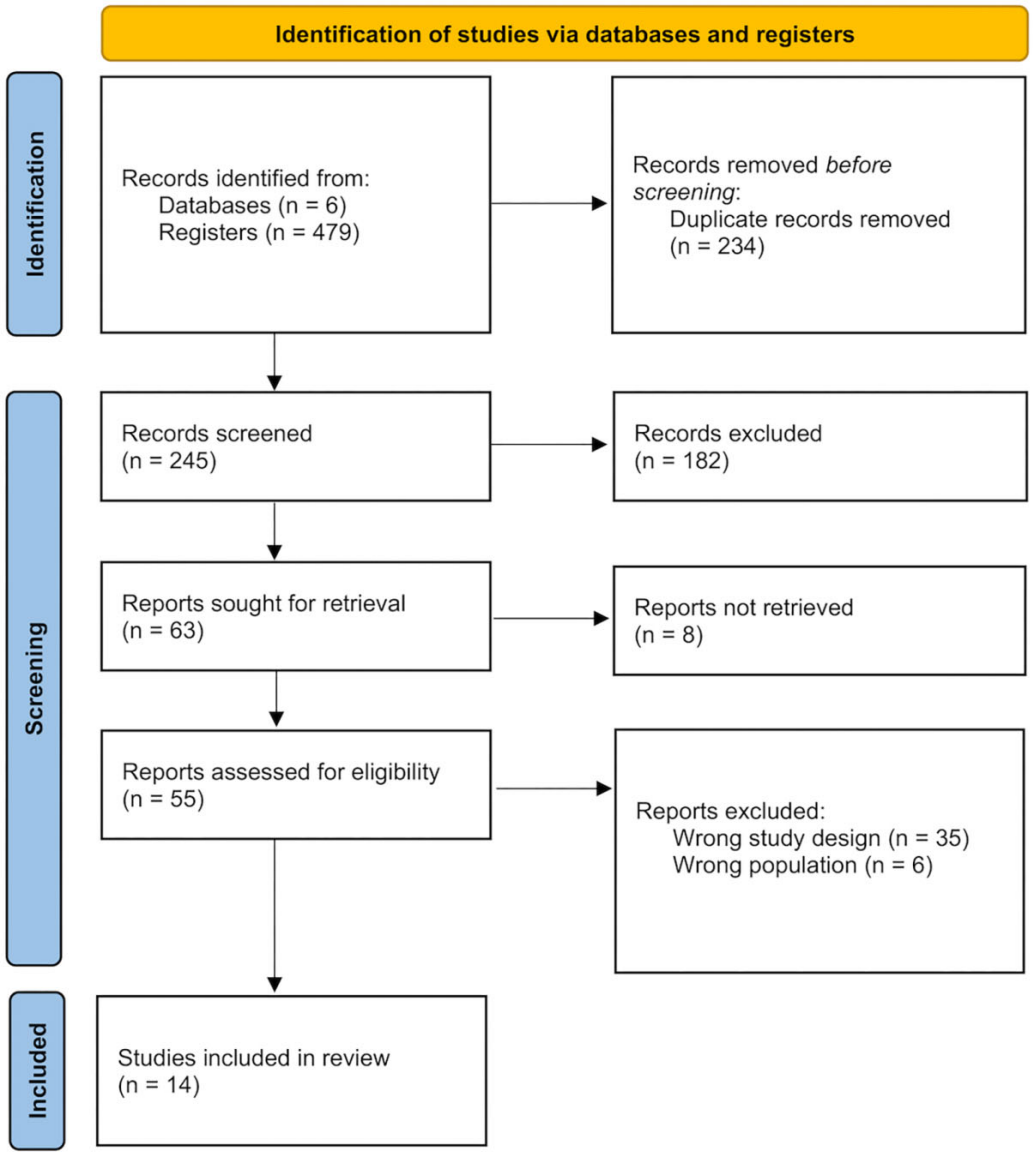
Prisma flow chart. This flow diagram illustrates the process of identifying screening, assessing eligibility and including studies in this systematic review and meta-analysis.

In our initial research, we identified 479 potential articles across seven databases. After removing 234 duplicate articles, we conducted screening based on title and abstract, leading to the exclusion of 182 articles. Eight of the 63 articles sought for retrieval were not retrieved, leaving 55 articles for eligibility assessment. These 55 articles underwent screening for eligibility, excluding 35 for wrong study designs and 6 for wrong population. Ultimately, 14 studies were included in this review. This process is summarized in our PRISMA flow diagram (Fig. 1).

### Characteristics of included studies

The cumulative sample size of the 14 included studies was 650 participants; all were adult patients undergoing maintenance hemodialysis for end-stage kidney disease (CKD stage 5D). Mean ages across studies ranged from 46 to 64 years, with most cohorts including patients in their fifth to sixth decade of life. Sex distribution was generally balanced but slightly male-predominant, with 34%–80% males and 20%–66% females, consistent with typical hemodialysis demographics. All studies enrolled clinically stable chronic hemodialysis patients; however, dialysis vintage was not reported, preventing classification into incident versus prevalent dialysis groups. The geographic distribution included countries such as Iran (35.7%), Egypt (21.4%), Taiwan (14.2%), the USA (7.14%), China (7.14%), Korea (7.14%) and Turkey (7.14%). The studies predominantly employed RCT designs (*n* = 11), with three crossover designs. All included studies evaluated vitamin C (ascorbic acid) supplementation delivered through different routes: 1 study administered ascorbic acid orally, 12 IV and 1 used both routes. Across studies, the frequency of vitamin C administration ranged from 3 to 30 times per month, with doses spanning from 1500 to 7500 mg monthly. Outcomes generally highlighted improved Hb levels (g/dL), TSAT (%) and EPO responsiveness, while reductions were observed in serum ferritin (ng/mL), TIBC (µg/dL), EPO dose (IU/week) and CRP levels (mg/L). Regarding Hb, 78.5% of studies (*n* = 11) reported an increase following vitamin C administration, 7.1% (*n* = 1) reported no significant effect and 14.2% (*n* = 2) did not assess this outcome. For TSAT, 71.4% of studies (*n* = 10) observed improvements, 14.2% (*n* = 2) reported no significant change and 14.2% (*n* = 2) did not evaluate this outcome. Serum ferritin levels decreased in 50% of studies (*n* = 7), while 14.2% (*n* = 2) found no significant difference and 35.7% (*n* = 5) did not report ferritin outcomes. Serum iron and TIBC were less consistently reported, with 35.7% of studies (*n* = 5) showing either increased serum iron or decreased TIBC, 14.2% (*n* = 2) reporting no significant change, and 50% (*n* = 7) not reporting these outcomes. We summarized this information in (Table 1) [24–37].

**Table 1:** Study characteristics.

Author, year	Country	Study design	Sample size	Follow-up (months)	Cases age, mean (SD)	Cases male (%)	Controls age, mean (SD)	Controls male (%)
El Shinnawy *et al*., 2021 [24]	Egypt	RCT	50	6	58.16 (8.41)	64	59.84 (6.39)	80
Tarng *et al*., 1998 [25]	Taiwan	RCT	24	2	54 (4)	58	56 (5)	67
Sedighi *et al*., 2013 [26]	Iran	RCT	40	3	57.1 (15.1)	40	60.1 (20.3)	45
Zhang *et al*., 2013 [27]	China	Crossover	100	3	64.3 (11.7)	34	64.4 (11.8)	58
Shahrbanoo *et al*., 2008 [28]	Iran	RCT	31	3	54.05 (3.2)	40	54.32 (4.1)	38
Tarng *et al*., 2004 [29]	Taiwan	RCT	36	0,23	55 (20)	38.9	59 (14)	44.4
Attallah *et al*., 2006 [30]	USA	RCT	42	6	50.6 (4.7)	45	49 (5.9)	45
Hajian *et al*., 2022 [31]	Iran	RCT	32	3	60.94 (14.29)	43.8	49.69 (21.82)	68.8
Jalalzadeh *et al*., 2012 [32]	Iran	Crossover	30	3	51.8 (16)	60	52.4 (14)	64
Behairy *et al*., 2021 [33]	Egypt	RCT	48	3	46.48 (15.57)	42	47.82 (16.74)	47
Bashardoust *et al*., 2017 [34]	Iran	RCT	39	2	54.29 (16.62)	58	52.20 (16.21)	60
El-Sharkawy *et al*., 2013 [35]	Egypt	RCT	60	2	46.46 (12.58)	NA	42.9 (13.98)	NA
Kang *et al*., 2012 [36]	Korea	RCT	58	3	54.5 (12.1)	45	56.9 (14.6)	48
Keven *et al*., 2003 [37]	Turkey	Crossover	60	6	42.0 (14.3)	43	40.2 (9.5)	47

NA: Not Available

### Risk of bias

The RoB 2.0 tool [20] was used to assess 11 randomized studies and three crossover studies. Within each study, the risk of bias was evaluated for each reported outcome. Two studies assessing Hb were identified as having a moderate risk of bias and another two were found to be high risk of bias. Two studies assessing EPO dose were found to have a moderate risk of bias, and one evaluating this outcome was found to be high risk of bias. Three studies evaluating ferritin were identified as moderate risk of bias, and one was deemed a high risk of bias. Two studies assessing serum iron were also related to having moderate risk of bias. For TSAT, two studies were found to have moderate risk of bias. In contrast, for TIBC one study was identified as having high risk of bias and two studies showed moderate risk of bias. CRP was assessed in two studies, both of which demonstrated a low risk of bias (Supplementary data, Fig. S1).

### Meta-analysis

#### Hb levels

**Figure 2:**
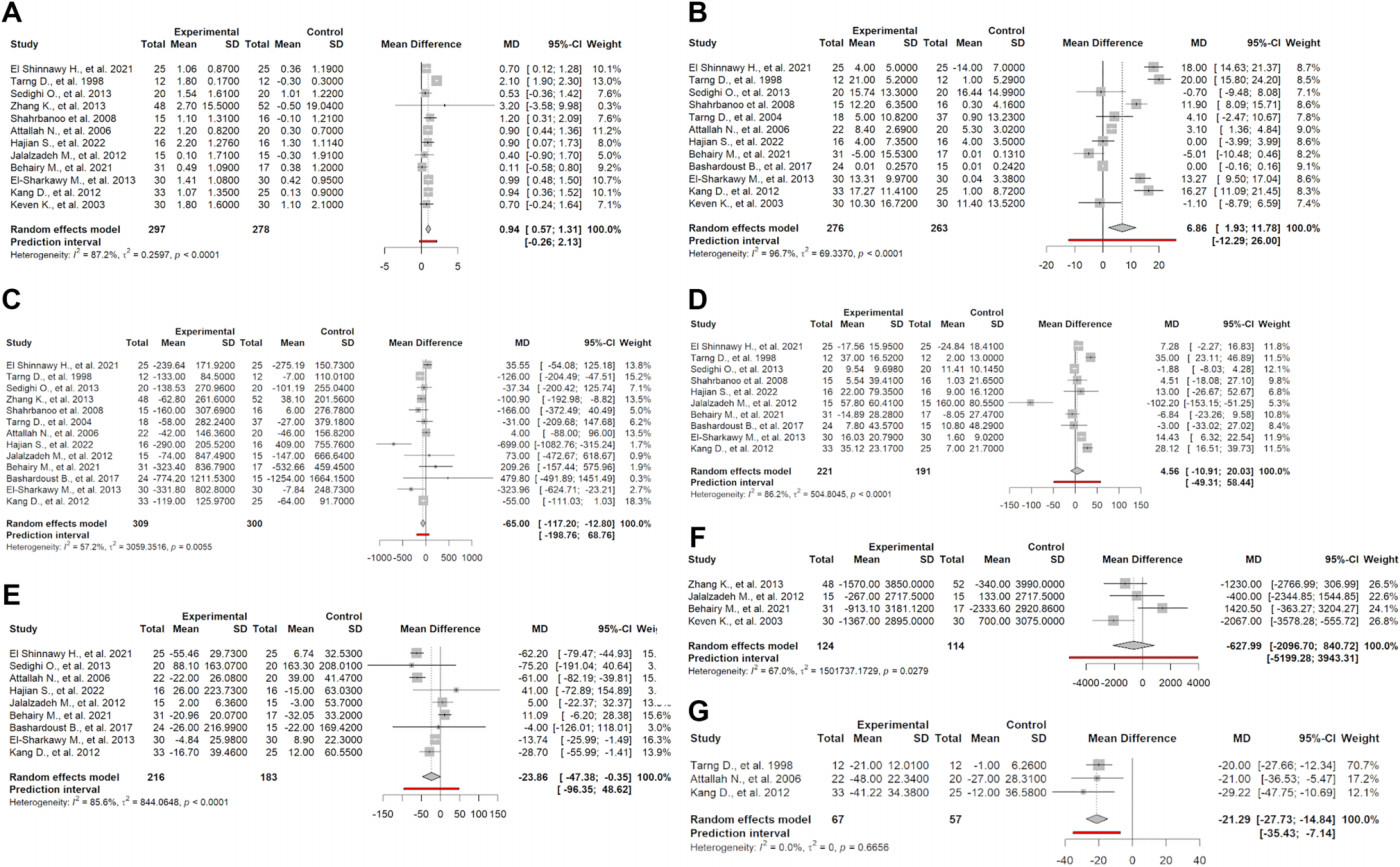
Forest plots meta-analysis. This forest plot displays pooled effect sizes with 95% CIs across included studies, evaluating the association of ascorbic acid supplementation with clinical outcomes. Squares represent effect sizes, and horizontal lines denote CIs: (**A**) Hb levels (g/dL); (**B**) TSAT (%); (**C**) Ferritin (ng/mL); (**D**) Iron level (µg/dL); (**E**) TIBC (µg/dL); (**F**) EPO dosage (units/week); (**G**) EPO dosage (units/kg/week).

**Figure 3:**
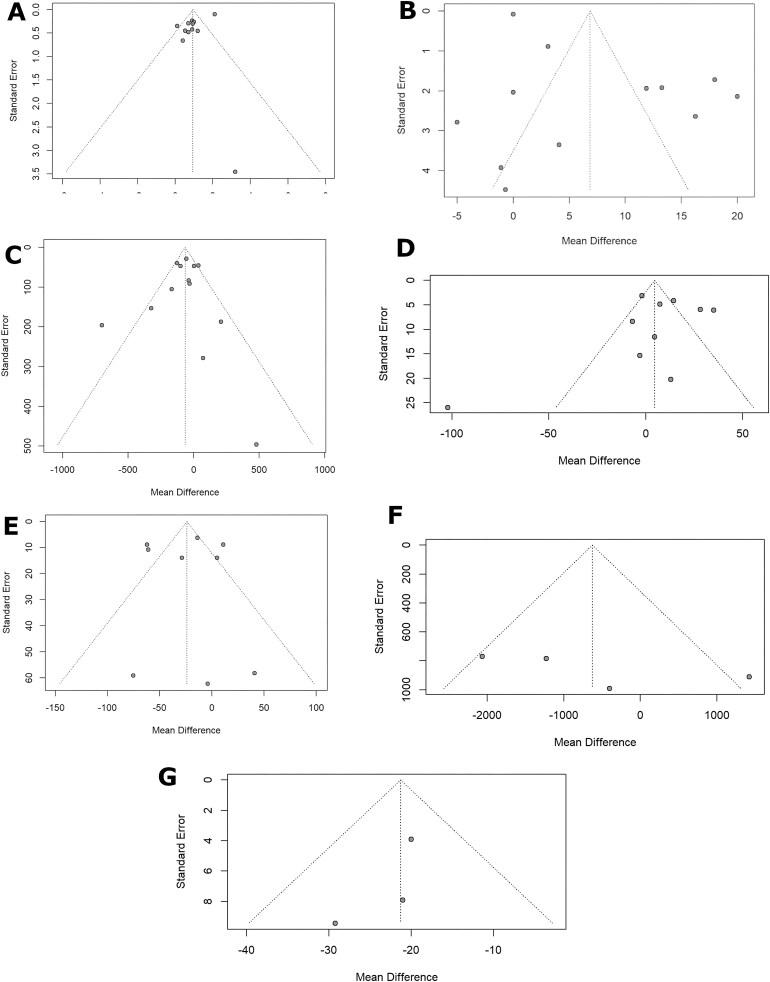
Funnel plots meta-analysis. These funnel plots assess publication bias for outcomes of ascorbic acid supplementation: (**A**) Hb levels (g/dL); (**B**) TSAT (%); (**C**) ferritin (ng/mL); (**D**) iron level (µg/dL); (**E**) TIBC (µg/dL); (**F**) EPO dosage (units/week); (**G**) EPO dosage (units/kg/week).

In this meta-analysis, we incorporated 12 studies [24–28, 30–33, 35–37] with a total sample size of 575 subjects, comprising 297 individuals treated with ascorbic acid and 278 in the control group. Using a random effects model, the pooled MD was estimated at 0.94 g/dL (95% CI 0.57 to 1.31; *P* < .01; *I*^2^ = 87.2%; Fig. 2A). The prediction interval ranged from −0.26 to 2.13. The funnel plot revealed asymmetry suggestive of potential publication bias (Fig. 3A). The Egger regression test was statistically significant for publication bias (*P* < .01). Subgroup analysis by route of administration demonstrated a statistically significant difference between IV and oral delivery (Supplementary data, Table S8). The IV subgroup (10 studies) showed a pooled MD of 1.02 (95% CI 0.65 to 1.39; *I*² = 87%), whereas the oral subgroup (2 studies) showed a smaller, non-significant effect MD 0.14 (95% CI –0.54 to 0.82; *I*² = 0.0%). Subgroups by risk of bias, study design, follow-up, dose, frequency/week, center type and year group showed no statistical significance. Influence analysis identified one study [25] as a significant influence on overall heterogeneity. Omitting this article, the revised MD is 0.79 (95% CI 0.58 to 1.00; *I*^2^ = 0.0%; Supplementary data, Table S9).

#### TSAT

We conducted a meta-analysis incorporating 12 studies [24–26, 28–31, 33–37] with a total sample size of 539 subjects, comprising 276 individuals treated with ascorbic acid and 263 in the control group. Using a random effects model, the pooled MD was estimated at 6.86 (95% CI 1.93 to 11.78; *P* < .01; *I*^2^ = 96.7%; Fig. 2B). The prediction interval ranged from −12.29 to 26.00. The Egger regression test was statistically significant for publication bias (*P* = .01). The funnel plot revealed asymmetry suggestive of potential publication bias (Fig. 3B). Subgroup analysis by route of administration showed a statistical significant difference (*P* < .01) between the IV route (11 studies) pooling an MD of 7.93 (95% CI 3.06 to 12.80; *I*^2^ = 97%) and the oral route (1 study), with an MD of –5.01 (95% CI –10.48 to 0.46), these results are to be interpreted with caution due to imbalance number of studies in each group (Supplementary data, Table S10). Subgroups by risk of bias, study design, follow-up, dose, frequency/week, center type and year group showed no statistical significance. Sensitivity analysis identified no studies as statistically influential (Supplementary data, Table S11).

#### Ferritin levels

We conducted a meta-analysis with 13 studies [24–36] with a total sample size of 609 subjects, comprising 309 individuals treated with ascorbic acid and 300 in the control group. Using a random effects model, the pooled MD was estimated at −65.00 ng/mL (95% CI −117.20 to −12.80; *P* = .01; *I*² = 57.2; Fig. 2C). The prediction interval ranged from −198.76 to 68.76. The funnel plot revealed asymmetry, suggestive of potential publication bias (Fig. 3C). The Egger regression test was not statistically significant for publication bias (*P* = .68). Subgroup analysis showed significant differences between groups by follow-up time (*P* = .04). (Supplementary data, Table S12). At 0.23 months, only one study was available, yielding a wide and imprecise estimate (MD −31.00; 95% CI −209.68 to 147.68) with no estimable heterogeneity. At 2 months, three studies showed a large and statistically significant reduction (MD −144.04; 95% CI −259.95 to −28.14; *I*² = 35.6%). At 3 months, seven studies also demonstrated a significant effect (MD −73.11; 95% CI −117.17 to − 29.05; *I*² = 58.6%). At 6 months, two studies reported a nonsignificant pooled estimate with a wide confidence interval (MD 20.19; 95% CI −44.01 to 84.39; *I*² = 0%). Subgroups by risk of bias, route of administration, study design, dose, frequency/week, center type and year group showed no statistical significance. Influence analysis did not identify any study as a major contributor to the heterogeneity (Supplementary data, Table S13).

#### Iron levels

We conducted a meta-analysis of 10 studies [24–26, 28, 31–36] including a total of 412 participants, with 221 receiving ascorbic acid and 191 assigned to the control group. Using a random effects model, the pooled MD was estimated at 4.56 µg/dL (95% CI −10.91 to 20.03; *P* = .56; *I*^2^ = 86.2%; Fig. 2D). The prediction interval ranged from −49.31.96 to 58.44. The Egger regression test was not statistically significant for publication bias (*P* = .71). The funnel plot revealed asymmetry suggestive of potential publication bias (Fig. 3D). Subgroup analysis by study design showed notable differences between RCTs and the single crossover study. Among the RCTs (nine studies), the pooled effect demonstrated a modest but imprecise increase of MD 10.99 (95% CI 0.79 to 21.20). In contrast, the crossover study (one study) showed a large and statistically significant reduction (MD –102.20; 95% CI –153.15 to –51.25). However, these results should be interpreted with caution given the substantial imbalance in the number of studies within each subgroup, which limits the reliability of between-group comparisons. Subgroups by risk of bias, route of administration, follow-up time, dose, frequency/week, center type and year group showed no statistical significance. Influence analysis identified this study [32] as influential (Supplementary data, Table S15). Excluding this study, reduced heterogeneity with a revised effect estimate is MD 11.00 µg/dL (95% CI 0.79 to 21.20; *I*^2^ = 83.0%).

#### TIBC

A meta-analysis with 9 studies [24, 26, 30–36] with a total sample size of 399 subjects, comprising 216 individuals treated with ascorbic acid and 183 in the control group. Using a random effects model, the pooled MD was estimated at −23.86 µg/dL (95% CI −47.38 to −0.35; *P* = .04; *I*^2^ = 85.6%; Fig. 2E). The prediction interval ranged from −96.35 to 48.62. The Egger regression test was not conducted due to a low number of studies. The funnel plot revealed asymmetry suggestive of potential publication bias (Fig. 3E). Subgroup analysis showed statistically significant differences across both the route of administration and follow-up duration (Supplementary data, Table S16). By route of administration, studies using IV therapy (eight studies) demonstrated a significant reduction in TIBC (MD –30.69; 95% CI –54.29 to –7.08; *I*² = 81%), whereas the single oral study showed a non-significant increase (MD 11.09; 95% CI –6.20 to 28.38). The between-group comparison was significant (*P* < .01). By follow-up time, the largest reduction was observed at 6 months (two studies) (MD –61.72; 95% CI –75.11 to –48.33; *I*² = 0%), followed by a moderate reduction at 2 months (two studies) (MD –13.64; 95% CI –25.83 to –1.45; *I*² = 0%). In contrast, the 3-month subgroup (five studies) showed a small, non-significant effect (MD –3.69; 95% CI –26.28 to 18.91; *I*² = 50.4%). Subgroups by risk of bias, dose, frequency/week, center type and year group showed no statistical significance. Influence analysis did not identify any study as a major contributor to heterogeneity (Supplementary data, Table S17).

#### EPO dosage

##### EPO dosage (units/week)

We conducted a meta‐analysis with four studies [27, 32, 33, 37] with a total sample size of 238 subjects, comprising 124 individuals treated with ascorbic acid and 114 in the control group. Using a random‐effects model, the pooled MD was estimated at –627.99 IU/week (95% CI −2096.70 to 840.72; *P* = .40; *I*² = 67.0% Fig. 2F). The prediction interval ranged from −5199.28 to 3943.31. The funnel plot revealed asymmetry suggestive of potential publication bias (Fig. 3F). The Egger regression test was not conducted due to a low number of studies.

Subgroup analysis revealed significant differences across risk of bias, study design and publication year. For risk of bias, studies judged as high risk (three studies) demonstrated a large reduction (MD –1360.64; 95% CI –2303.23 to –418.05; *I*² = 0%), whereas the single low-risk of bias study showed an opposite, non-significant effect (MD 1420.50; 95% CI –363.27 to 3204.27). In the subgroup analysis by study design, crossover trials (three studies) showed a substantial decrease in dose (MD –1360.64; 95% CI –2303.23 to –418.05; *I*² = 0%), while the only RCT demonstrated a non-significant increase (MD 1420.50; 95% CI –363.27 to 3204.27). When stratified by year group, studies published before 2015 (three studies) also showed a significant reduction (MD –1360.64; 95% CI –2303.23 to –418.05; *I*² = 0%), whereas the single study published after 2015 again showed a non-significant increase (MD 1420.50; 95% CI –363.27 to 3204.27). These results should be interpreted with caution given the imbalance in the number of studies contributing to each subgroup. Subgroups by dose, frequency/week and center type, follow-up and route of administration showed no statistical significance. Influence analysis identified these studies [33, 37] as influential. Omitting these articles, the revised MD is –910.91 (95% CI –2116.79 to 294.97; *I*^2^ = 0.0%).

#### EPO dosage (units/kg/week)

We conducted a meta‐analysis with three studies with a total sample size of 124 subjects, comprising 67 individuals treated with ascorbic acid and 57 in the control group. Using a random effects model, the pooled MD was estimated at –21.29 IU/kg/week (95% CI −27.73 to 14.84; *P* < .01; *I*² = 0.0%; Fig. 2G). The prediction interval ranged from −35.43 to –7.14. The funnel plot revealed asymmetry, suggesting potential publication bias (Fig. 3G). The Egger regression test was not conducted due to a low number of studies.

### Assessment of the certainty of the evidence and summary of findings

Across outcomes, the certainty of evidence ranged from low to very low, mainly due to substantial inconsistency, serious imprecision and suspected publication bias. Hb and ferritin showed low certainty, downgraded primarily for heterogeneity and potential reporting bias, while TSAT, iron levels, TIBC and EPO dose (units/week) demonstrated very low certainty because their estimates were highly heterogeneous, crossed thresholds of clinical importance, and suggested possible publication bias. EPO dose (units/kg/week) showed low certainty, downgraded for imprecision and suspected publication bias. Overall, confidence in the effect estimates is limited, and additional well-designed randomized trials are needed to clarify these findings (Supplementary data, Table S2).

## DISCUSSION

This systematic review and meta-analysis evaluated the hematologic and iron-related effects of ascorbic acid supplementation compared with placebo or standard therapy in patients with CKD undergoing hemodialysis. By including 11 RCTs and 3 crossover trials with 650 participants, this review improves statistical power and addresses limitations of prior studies [27, 32, 35, 37] which were constrained by small sample sizes, narrow geographic scope and inconsistent follow-up durations. This review followed PRISMA guidelines, was registered in PROSPERO and used Cochrane’s RoB 2.0 tool to ensure methodological rigor.

Although clinical guidelines such as KDIGO and the Kidney Disease Outcomes Quality Initiative (KDOQI) recommend EPO and iron supplementation as the foundation of CKD-related anemia management, they currently do not endorse vitamin C due to insufficient and inconsistent evidence [7]. Our findings suggest outcome-specific effects. Hb levels showed a statistically and clinically significant increase (MD 0.94 g/dL; *P* < .01), while TSAT improved (MD 6.861%; *P* < .01). In addition, TIBC decreased significantly (MD −23.86 µg/dL; *P* = .03) and ferritin levels fell modestly (MD −65.00 ng/mL; *P* = .04). By contrast, serum iron levels remain unchanged (*P* = .56); altogether, these findings may be explained by improved release and utilization of stored iron, which in turn promotes more efficient erythropoiesis. EPO dose requirements (U/week) were not significantly reduced (*P* = .40); however, in our analysis of three studies that assessed EPO dosage requirements in (U/kg/week) there was a modest decrease in EPO dose requirements (MD –21.29 IU/kg/week; *P* ≤ .01); this discrepancy is likely explained by differences in variability were absolute dosing is influenced by patient body weight introducing substantial heterogeneity, whereas weight-adjusted dosing standardizes EPO requirements across individuals revealing a more consistent treatment effect; thus the significant findings in U/kg/week likely reflect a more biologically meaningful measure of EPO responsiveness. The GRADE evaluation revealed low‐certainty evidence for changes in Hb, ferritin and EPO dose (units/kg/week), and very low certainty for TSAT, serum iron, TIBC and EPO dose expressed in units/week. These judgments reflected notable inconsistency across studies, wide CIs indicating imprecision and concerns about potential publication bias. Taken together, these findings support the interpretation that ascorbic acid may exert its effect primarily by enhancing iron mobilization and utilization rather than by increasing total circulating iron, although the overall confidence in these estimates remains limited.

Although short-term IV vitamin C appears generally well tolerated in hemodialysis patients, none of the included trials systematically assessed long-term adverse events, particularly oxalate accumulation. As reported previously [38], even moderate doses can increase plasma oxalate, highlighting that while acute administration seems safe, the long-term safety of repeated supplementation remains uncertain. Therefore, clinical enthusiasm should be tempered, and careful monitoring is warranted if therapy is extended. Subgroup analyses indicated that IV vitamin C consistently improved Hb and TSAT, whereas oral dosing showed smaller effects, suggesting that route of administration is the most important clinically relevant determinant of hematologic response in hemodialysis patients. Yet these findings should be interpreted cautiously, given the small number of studies in each subgroup. Sensitivity analyses identified certain influential trials, such as one crossover trial with a total sample size of 30 and a 3-month follow-up period [32]; in this study, vitamin C was administered IV three times per month, for a total monthly dose of 1500 mg. The sensitivity analysis for iron levels showed an effect estimate of 14.827 (95% CI –2.707 to 32.361), with *I*² = 0.935, DFFITS = –1.2600, Cook’s D = 0.6989 and QE (del) = 61.62. This influential effect was primarily driven by this study [25], which reported a marked increase in iron levels following high-dose IV vitamin C administration. The influential contribution reflects its higher IV dosing schedule, its inclusion of patients with pronounced functional iron deficiency and EPO hyperresponsiveness, and its large reduction in ferritin relative to other trials. These characteristics differed from later lower-dose or oral studies and contributed to the heterogeneity observed in ferritin subgroup analyses but removing them did not eliminate the overall heterogeneity. Funnel plots revealed asymmetry, suggesting small-study effects, with some outcomes reflecting a significant Egger’s regression suggestive for publication bias. These influential trials affected the pooled results primarily due to their unique characteristics. For example, studies with higher IV doses or patients with pronounced functional iron deficiency contributed disproportionately to improvements in Hb and TSAT, thereby increasing heterogeneity in these outcomes. Their dosing schedules, baseline patient characteristics and study durations differed from other trials, highlighting why these studies appeared as outliers in sensitivity analyses. Recognizing these influential trials helps contextualize the variability in pooled estimates and underscores that observed effects may be partly driven by specific study conditions rather than uniform effects across all included trials. Although some funnel plots displayed visual asymmetry, Egger’s regression tests were non-significant for some of the outcomes. This apparent discrepancy may be due to the relatively small number of studies included in each analysis, heterogeneity in dosing regimens, routes of administration and patient characteristics, or the disproportionate influence of specific trials with higher IV doses. While these findings do not definitively indicate publication bias, they highlight the need for cautious interpretation of pooled estimates and underscore the importance of larger, well-powered studies to confirm these results.

Previous meta-analyses have reported effects of vitamin C supplementation on anemia-related outcomes in hemodialysis patients, but their scope and methodological rigor were limited. This study observed improvements in Hb and TSAT with IV vitamin C [39]; however, the included studies were small, had short follow-up, and reported limited safety data, which restricted confidence in the findings. The more recent review [40], evaluated vitamin C co-administered with iron across various populations and highlighted inconsistent benefits for Hb, ferritin and EPO dose reduction, noting heterogeneity in study design, dosing regimens and duration of follow-up.

Compared with these prior reviews, our meta-analysis includes a larger pooled sample (14 studies with 650 participants) and incorporated more recent trials, allowing for outcome-specific subgroup analyses by route, dose and frequency. This provides a more nuanced understanding of potential effect modifiers. For example, we observed that thrice-weekly IV vitamin C consistently improved TSAT and Hb compared with less frequent dosing, a finding not addressed in earlier reviews. In addition, sensitivity analyses in our study help clarify the influence of trials with high IV doses or patients with functional iron deficiency, adding context to sources of heterogeneity noted in previous meta-analyses. Overall, our findings corroborate the hematologic benefits reported previously while offering greater granularity regarding administration strategies and mechanistic insights. Yet, the absence of patient-centered outcomes such as quality of life, functional status, hospitalization or mortality in the included trials prevent translation of these biochemical improvements into confirmed clinical benefits.

The observed increases in Hb and TSAT, despite minimal effects on serum iron and EPO dose, are consistent with vitamin C’s established role in iron mobilization rather than expansion of total iron stores. Ascorbic acid facilitates the reduction of ferric (Fe^3+^) to ferrous (Fe^2+^) iron, a critical step that enhances intestinal absorption, improves solubility of transferrin-bound iron, and supports iron transport across cellular membranes [41]. In hemodialysis patients with functional iron deficiency, vitamin C also promotes release of iron from the reticuloendothelial system, thereby increasing the pool of bioavailable iron for erythropoiesis without necessarily increasing total body iron [29]. Additionally, vitamin C acts as a cofactor for ferroxidase and iron-regulatory enzymes, supporting efficient incorporation of iron into Hb synthesis and reducing iron sequestration mediated by inflammation-driven pathways such as hepcidin upregulation [42]. These mechanisms explain the consistent reductions in ferritin and increase in TSAT observed in our analysis, reflecting improved iron mobilization rather than iron overload, and help clarify why vitamin C’s biochemical effects may occur even in the absence of significant changes in serum iron concentrations.

The findings highlight biologic plausibility but also underscore substantial limitations, including high heterogeneity, possible publication bias, modest effect sizes, short-term follow-up and absent patient-centered outcomes. A key limitation is that none of the included trials reported dialysis vintage, preventing subgroup or sensitivity analyses based on whether participants were newly initiated or chronically treated hemodialysis patients.

Currently clinical practice guidelines recommend ESAs combined with iron supplementation as the definitive standard for CKD-related anemia. Given its low cost and safety in short-term trials, vitamin C may represent a potential adjunctive therapy for anemia in selected patients, with significant improvements in Hb, TSAT and TIBC; however, current evidence remains insufficient to warrant changes in guideline recommendations. Large, multicenter RCTs with standardized dosing regimens, extended follow-up, rigorous safety monitoring and patient-centered outcomes are needed to establish whether these biochemical benefits translate into meaningful clinical improvements.

## CONCLUSION

This systematic review and meta-analysis demonstrates that ascorbic acid supplementation produces modest improvements in key hematologic and iron-related parameters in hemodialysis patients with anemia, including significant increases in Hb and TSAT, and reductions in ferritin and TIBC. These findings are biologically possible and consistent with vitamin C’s role in enhancing iron mobilization and utilization rather than increasing circulating iron stores.

The certainty of evidence across outcomes ranged from low to very low, largely due to substantial heterogeneity, wide CIs and suspected publication bias. Although vitamin C supplementation, administered orally or IV at doses of 1500–7500 mg and frequencies of 3–30 administrations per month, appears to increase Hb and TSAT, and to modestly reduce ferritin levels, these findings are based on low or very low certainty, limiting confidence in their clinical relevance. Importantly, effects on serum iron concentrations and absolute EPO dose remain highly uncertain, with very low certainty due to serious inconsistency and imprecision. Although the weight-adjusted EPO dose showed a modest and statistically significant reduction, the certainty of evidence was low, and the clinical relevance remains unclear.

Therefore, despite potential biochemical improvements, the overall evidence remains insufficient to support the routine use of vitamin C for anemia management in hemodialysis patients. Future well-designed, adequately powered randomized trials with standardized dosing regimens, longer follow-up, comprehensive safety assessment, including oxalate monitoring, and incorporation of patient-centered outcomes are needed to determine whether these biochemical improvements translate into durable and clinically meaningful benefits (Table 2).

**Table 2:** Meta-analysis outcomes.

Outcome	Hb (g/dL)	TSAT (%)	Ferritin (ng/mL)	Serum iron (µg/dL)	TIBC (µg/dL)	EPO dosage (IU/week)	EPO dosage (units/kg/week)
No. of studies	12	12	13	10	9	4	3
No. total of participants	575	539	609	412	399	238	124
Effect size [MD (95% CI)]	0.94 (0.57 to 1.31)	6.86 (1.93 to 11.78)	−65.00 (−117.20 to −12.80)	4.56 (−10.91 to 20.03)	−23.86 (−47.38 to −0.35)	–627.99 (−2096.70 to 840.72)	–21.29 (−27.73 to 14.84)
*I*² (%)	87.2	96.7	57.2	86.2	85.6	67.0	0.0
*P*-value	<.01	<.01	.01	.56	.04	.40	<.01

## Supplementary Material

sfag070_Supplemental_File

## Data Availability

All the data used to perform this analysis is publicly available in the referenced articles. The rest of the material is at the author’s disposition upon reasonable request.
