# Supplementary material for: Ascorbic acid as an adjunct therapy for anemia in erythropoietin-treated hemodialysis patients: a systematic review and meta-analysis
Source: Clin Kidney J. 2026 Apr 22;19(6):sfag070. doi: 10.1093/ckj/sfag070 (PMC13232050; doi:10.1093/ckj/sfag070)
Supplement: sfag070_Supplemental_File [file sfag070_supplemental_file.docx]

**Methodology for supplementary material.**

***Searching methods***

A systematic search was initially conducted on May 17, 2025 on Pubmed/MEDLINE, Cochrane, Scopus, Web of Science, EMBASE, CINDAHL, and Google scholar using the following terms: All detailed search strategies can be found in the supplementary material (Supplementary Tables 1-7)

***Data extraction***

Two independent reviewers extracted the data (E.C.M. and Y.C.C.), and disagreements were resolved by consensus. When multiple overlapping reports from the same study were identified, the information from the one containing the most relevant information or the first published report was included. Extracted data included sample sizes, intervention types, and measured outcomes.  Conventional methods were used for data extraction, complemented by specialized tools such as WebPlotDigitizer (Automeris, Austin, TX, USA)​^12^​ for digitizing data from graphs, Cochrane Calculator​^13^​ for statistical conversions, and StatsToDo​^14^​ for advanced calculations. These outcomes compromised hemoglobin, serum ferritin, serum iron, erythropoietin dose, TSAT, TIBC and CRP. Additional extracted data included subgroup characteristics, such as country of study, risk of bias levels and study design.

***Assessment of risk of bias in included studies***

To assess the quality of the studies included in the systematic review, according to the Cochrane guidelines we applied the Cochrane RoB 2.0 tool​^15^​ for randomized controlled trials (RCTs). Two independent reviewers (A.C.L and M.V.S.) evaluated the risk of bias in each study, considering the specific criteria and guidelines provided by the respective tools. Any reviewer discrepancies were resolved through discussion with a third, blinded reviewer (S.A.B).

***Statistical analysis***

A meta-analysis was performed using R version 3.4.3 (R Core Team) with the meta and metafor packages​^16,17^​. The pooled effect of the outcomes was examined using a random-effects meta-analysis (DerSimonian-Laird approach). Whenever the number of studies reporting an outcome of interest was insufficient, only a qualitative analysis of the results was performed. Effect sizes were expressed as relative risk (RR), mean difference (MD), or standardized mean difference (SMD) with a 95% confidence interval. The I² statistic assessed heterogeneity, and the following cut-off values were used for interpretation: <25%, 25–50%, and >50% were considered small, medium, and large heterogeneity, respectively. For all outcomes, sensitivity analyses using the leave-one-out method were performed to determine the influence of individual studies on the overall effect.  Egger's regression test was used to examine publication bias when 10 or more reports with the same outcome were available. Whenever possible, subgroup analyses were planned based on risk of bias and performed in primary outcomes.

**Supplementary material index.**

Supplementary table 1. Search in PubMed 5

Supplementary table 2. Search in Embase 5

Supplementary table 3. Search in Web of Science 6

Supplementary table 4. Search in Scopus 6

Supplementary table 5. Search in CINAHL 6

Supplementary table 6. Search in Google scholar 7

Supplementary table 7. Search in Cochrane 7

Supplementary Table 8. Subgroup analysis: hemoglobin levels. 8

Supplementary Table 9. Influence analysis: Hemoglobin levels 9

Supplementary Table 10. Subgroup analysis: TSAT 10

Supplementary Table 11. Influence analysis: TSat levels 11

Supplementary Table 12. Subgroup analysis: Ferritin 12

Supplementary Table 13. Influence analysis: Ferritin 13

Supplementary Table 14. Subgroup analysis: Iron levels. 13

Supplementary Table 15. Sensitivity analysis: Iron levels 14

Supplementary Table 16. Subgroup analysis: TIBC 15

Supplementary Table 17. Influence analysis: TIBC levels 16

Supplementary Table 18. Subgroup analysis: EPO dosage Units/Week 16

Supplementary table 19. Influence analysis: EPO dosage Units/ Week 17

Supplementary table 20. Assessment of the certainty of the evidence and summary of findings 18

Supplementary figure 1. Risk of bias assessment 19

PRISMA 2020 checklist. 19

PRISMA 2020 Abstract checklist. 23

Supplementary table 1. Search in PubMed

| Boolean search | Results |
| --- | --- |
| ((Ascorbic Acid [Mesh]) OR (ascorbic acid [Title/Abstract]) OR (vitamin C [Title/Abstract]) OR (L-ascorbic acid [Title/Abstract]) OR (ascorbate [Title/Abstract]) OR (ascorbic supplementation [Title/Abstract]) OR (vitamin C supplementation [Title/Abstract]) OR (vitamin C therapy [Title/Abstract]))  AND((Anemia, Iron-Deficiency [Mesh]) OR( iron deficiency anemia [Title/Abstract]) OR (iron-deficiency anemia [Title/Abstract]) OR (IDA [Title/Abstract]) OR (anemia [Title/Abstract]) OR (anaemia [Title/Abstract]) OR (nutritional anemia [Title/Abstract]) OR (iron-deficient [Title/Abstract]))  AND  ((Erythropoietin [Mesh]) OR (erythropoietin [Title/Abstract]) OR (recombinant human erythropoietin [Title/Abstract]) OR (rhEPO [Title/Abstract]) OR (EPO [Title/Abstract]) OR (ESA [Title/Abstract]) OR (erythropoiesis stimulating agent [Title/Abstract]) OR (erythropoiesis-stimulating agents [Title/Abstract]) OR (epoetin alfa [Title/Abstract]) OR (epoetin beta [Title/Abstract]) OR (darbepoetin alfa [Title/Abstract]))  AND  ((Renal Dialysis [Mesh]) OR (hemodialysis [Title/Abstract]) OR (haemodialysis [Title/Abstract]) OR (dialysis [Title/Abstract]) OR (renal replacement therapy [Title/Abstract]) OR (RRT [Title/Abstract]) OR (chronic kidney disease stage 5 [Title/Abstract]) OR (CKD5 [Title/Abstract]) OR (end-stage renal disease [Title/Abstract]) OR (ESRD [Title/Abstract])) | 57 |

Supplementary table 2. Search in Embase

| Boolean search | Results |
| --- | --- |
| ('ascorbic acid'/exp OR 'ascorbic acid':ti,ab OR 'vitamin c':ti,ab OR 'l-ascorbic acid':ti,ab OR ascorbate:ti,ab OR 'ascorbic supplementation':ti,ab OR 'vitamin c supplementation':ti,ab OR 'vitamin c therapy':ti,ab)  AND  ('iron deficiency anemia'/exp OR 'iron deficiency anemia':ti,ab OR 'iron-deficiency anemia':ti,ab OR ida:ti,ab OR anemia:ti,ab OR anaemia:ti,ab OR 'nutritional anemia':ti,ab OR 'iron-deficient':ti,ab)  AND  ('erythropoietin'/exp OR erythropoietin:ti,ab OR 'recombinant human erythropoietin':ti,ab OR rhepo:ti,ab OR epo:ti,ab OR esa:ti,ab OR 'erythropoiesis stimulating agent':ti,ab OR 'erythropoiesis-stimulating agents':ti,ab OR 'epoetin alfa':ti,ab OR 'epoetin beta':ti,ab OR 'darbepoetin alfa':ti,ab)  AND  ('renal dialysis'/exp OR hemodialysis:ti,ab OR haemodialysis:ti,ab OR dialysis:ti,ab OR 'renal replacement therapy':ti,ab OR rrt:ti,ab OR 'chronic kidney disease stage 5':ti,ab OR ckd5:ti,ab OR 'end-stage renal disease':ti,ab OR esrd:ti,ab) | 125 |

Supplementary table 3. Search in Web of Science

| Boolean search | Results |
| --- | --- |
| TS=("ascorbic acid" OR "vitamin C" OR "L-ascorbic acid" OR ascorbate OR "ascorbic supplementation" OR "vitamin C supplementation" OR "vitamin C therapy")  AND  TS=("iron deficiency anemia" OR "iron-deficiency anemia" OR IDA OR anemia OR anaemia OR "nutritional anemia" OR "iron-deficient")  AND  TS=(erythropoietin OR "recombinant human erythropoietin" OR rhEPO OR EPO OR ESA OR "erythropoiesis stimulating agent" OR "erythropoiesis-stimulating agents" OR "epoetin alfa" OR "epoetin beta" OR "darbepoetin alfa")  AND  TS=(hemodialysis OR haemodialysis OR dialysis OR "renal replacement therapy" OR RRT OR "chronic kidney disease stage 5" OR CKD5 OR "end-stage renal disease" OR ESRD) | 95 |

Supplementary table 4. Search in Scopus

| Boolean search | Results |
| --- | --- |
| (TITLE-ABS("ascorbic acid") OR TITLE-ABS("vitamin C") OR TITLE-ABS("L-ascorbic acid") OR TITLE-ABS(ascorbate) OR TITLE-ABS("ascorbic supplementation") OR TITLE-ABS("vitamin C supplementation") OR TITLE-ABS("vitamin C therapy"))  AND  (TITLE-ABS("iron deficiency anemia") OR TITLE-ABS("iron-deficiency anemia") OR TITLE-ABS(IDA) OR TITLE-ABS(anemia) OR TITLE-ABS(anaemia) OR TITLE-ABS("nutritional anemia") OR TITLE-ABS("iron-deficient"))  AND  (TITLE-ABS(erythropoietin) OR TITLE-ABS("recombinant human erythropoietin") OR TITLE-ABS(rhEPO) OR TITLE-ABS(EPO) OR TITLE-ABS(ESA) OR TITLE-ABS("erythropoiesis stimulating agent") OR TITLE-ABS("erythropoiesis-stimulating agents") OR TITLE-ABS("epoetin alfa") OR TITLE-ABS("epoetin beta") OR TITLE-ABS("darbepoetin alfa"))  AND  (TITLE-ABS(hemodialysis) OR TITLE-ABS(haemodialysis) OR TITLE-ABS(dialysis) OR TITLE-ABS("renal replacement therapy") OR TITLE-ABS(RRT) OR TITLE-ABS("chronic kidney disease stage 5") OR TITLE-ABS(CKD5) OR TITLE-ABS("end-stage renal disease") OR TITLE-ABS(ESRD)) | 46 |

Supplementary table 5. Search in CINAHL

| Boolean search | Results |
| --- | --- |
| ((MH "Ascorbic Acid+") OR TI "ascorbic acid" OR AB "ascorbic acid" OR TI "vitamin C" OR AB "vitamin C" OR TI "L-ascorbic acid" OR AB "L-ascorbic acid" OR TI ascorbate OR AB ascorbate OR TI "ascorbic supplementation" OR AB "ascorbic supplementation" OR TI "vitamin C supplementation" OR AB "vitamin C supplementation" OR TI "vitamin C therapy" OR AB "vitamin C therapy")  AND  ((MH "Anemia, Iron-Deficiency+") OR TI "iron deficiency anemia" OR AB "iron deficiency anemia" OR TI "iron-deficiency anemia" OR AB "iron-deficiency anemia" OR TI IDA OR AB IDA OR TI anemia OR AB anemia OR TI anaemia OR AB anaemia OR TI "nutritional anemia" OR AB "nutritional anemia" OR TI "iron-deficient" OR AB "iron-deficient")  AND  ((MH "Erythropoietin+") OR TI erythropoietin OR AB erythropoietin OR TI "recombinant human erythropoietin" OR AB "recombinant human erythropoietin" OR TI rhEPO OR AB rhEPO OR TI EPO OR AB EPO OR TI ESA OR AB ESA OR TI "erythropoiesis stimulating agent" OR AB "erythropoiesis stimulating agent" OR TI "erythropoiesis-stimulating agents" OR AB "erythropoiesis-stimulating agents" OR TI "epoetin alfa" OR AB "epoetin alfa" OR TI "epoetin beta" OR AB "epoetin beta" OR TI "darbepoetin alfa" OR AB "darbepoetin alfa")  AND  ((MH "Renal Dialysis+") OR TI hemodialysis OR AB hemodialysis OR TI haemodialysis OR AB haemodialysis OR TI dialysis OR AB dialysis OR TI "renal replacement therapy" OR AB "renal replacement therapy" OR TI RRT OR AB RRT OR TI "chronic kidney disease stage 5" OR AB "chronic kidney disease stage 5" OR TI CKD5 OR AB CKD5 OR TI "end-stage renal disease" OR AB "end-stage renal disease" OR TI ESRD OR AB ESRD) | 10 |

Supplementary table 6. Search in Google scholar

| Boolean search | Results |
| --- | --- |
| ("ascorbic acid" OR "vitamin C" OR "ascorbic supplementation" OR "vitamin C supplementation") AND ("iron deficiency anemia" OR anemia ) AND (erythropoietin OR rhEPO OR EPO) AND (hemodialysis OR haemodialysis OR dialysis) | 100 |

Supplementary table 7. Search in Cochrane

| Line | Boolean search | Results |
| --- | --- | --- |
| #1 | MeSH descriptor: [Ascorbic Acid] explode all trees | 2802 |
| #2 | "ascorbic acid":ti,ab,kw OR "vitamin C":ti,ab,kw OR "L-ascorbic acid":ti,ab,kw OR ascorbate:ti,ab,kw OR "ascorbic supplementation":ti,ab,kw OR "vitamin C supplementation":ti,ab,kw OR "vitamin C therapy":ti,ab,kw | 7057 |
| #3 | #1 OR #2 | 7059 |
| #4 | MeSH descriptor: [Anemia, Iron-Deficiency] explode all trees | 1813 |
| #5 | "iron deficiency anemia":ti,ab,kw OR "iron-deficiency anemia":ti,ab,kw OR IDA:ti,ab,kw OR anemia:ti,ab,kw OR anaemia:ti,ab,kw OR "nutritional anemia":ti,ab,kw OR "iron-deficient":ti,ab,kw | 26713 |
| #6 | #4 OR #5 | 26713 |
| #7 | MeSH descriptor: [Erythropoietin] explode all trees | 2622 |
| #8 | erythropoietin:ti,ab,kw OR "recombinant human erythropoietin":ti,ab,kw OR rhEPO:ti,ab,kw OR EPO:ti,ab,kw OR ESA:ti,ab,kw OR "erythropoiesis stimulating agent":ti,ab,kw OR "erythropoiesis-stimulating agents":ti,ab,kw OR "epoetin alfa":ti,ab,kw OR "epoetin beta":ti,ab,kw OR "darbepoetin alfa":ti,ab,kw | 6816 |
| #9 | #7 OR #8 | 6816 |
| #10 | MeSH descriptor: [Renal Dialysis] explode all trees | 7401 |
| #11 | hemodialysis:ti,ab,kw OR haemodialysis:ti,ab,kw OR dialysis:ti,ab,kw OR "renal replacement therapy":ti,ab,kw OR RRT:ti,ab,kw OR "chronic kidney disease stage 5":ti,ab,kw OR CKD5:ti,ab,kw OR "end-stage renal disease":ti,ab,kw OR ESRD:ti,ab,kw | 28624 |
| #12 | #10 OR #11 | 28656 |
| #13 | #3 AND #6 AND #9 AND #12 | 46 |

Supplementary Table 8. Subgroup analysis: hemoglobin levels.

| **Variable** | **Subgroup** | **k** | **MD (95% CI)** | **I² (%)** | **p-value (Between Groups)** |
| --- | --- | --- | --- | --- | --- |
| Risk of bias | Low | 7 | 0.9339 (0.3524 to 1.5155) | 93.1 | 0.8129 |
|  | High | 4 | 0.9301 (0.5815 to 1.2788) | 0.0 |  |
|  | Some Concerns | 1 | 1.2000 (0.310 to 2.0894) | - |  |
| Route of administration | IV | 10 | 1.0248 [0.6577; 1.3918] | 87.0 | 0.0257 |
|  | Oral | 2 | 0.1414 [-0.5426; 0.8254] | 0.0 |  |
| Study design | RCT | 9 | 0.9778 [0.5700; 1.3855] | 90.0 | 0.4271 |
|  | Cross-over | 3 | 0.6286 [-0.1303; 1.3875] | 0.0 |  |
| Follow-up | 6 | 3 | 0.8069 [0.4706; 1.1432] | 0.0 | 0.3299 |
|  | 2 | 2 | 1.5716 [0.4851; 2.6581] | 93.6 |  |
|  | 3 | 7 | 0.7032 [0.3444; 1.0621] | 0.0 |  |
| Dose of Ascorbic Acid a month (mg) | <2000 | 2 | 0.6503 [0.1225; 1.1782] | 0.0 | 0.1415 |
|  | 2000–5000 | 4 | 1.1940 [0.4990; 1.8889] | 92.7 |  |
|  | >5000 | 4 | 0.8901 [0.4661; 1.3140] | 0.0 |  |
|  | <5000 | 1 | 0.1100 [-0.5775; 0.7975] | -- |  |
| Frequency/Week | 3 times -first week | 2 | 0.6503 [0.1225; 1.1782] | 0.0 | 0.6138 |
|  | 3 | 7 | 1.0044 [0.5105; 1.4984] | 91.3 |  |
|  | 2 | 1 | 0.5300 [-0.3553; 1.4153] | -- |  |
|  | 7 | 1 | 3.2000 [-3.5829; 9.9829] | -- |  |
| Center type | Single Center | 7 | 0.9594 [0.4539; 1.4649] | 91.7 | 0.7063 |
|  | Multi-Center | 4 | 0.8330 [0.4129; 1.2531] | 0.0 |  |
| Year Group | After 2015 | 3 | 0.5540 [0.1176; 0.9904] | 20.5 | 0.1273 |
|  | Pre 2015 | 8 | 1.0581 [0.5790; 1.5371] | 87.6 |  |

Supplementary Table 9. Influence analysis: Hemoglobin levels

| **Author** | **Effect (95% CI: Lower; Upper)** | **I^2** | **DFFITS** | **Cook's D** | **QE (del)** | **Is Influential** |
| --- | --- | --- | --- | --- | --- | --- |
| El Shinnawy H., et al. 2021 | 0.961 (95% CI: 0.562; 1.361) | 0.872 | -0.1156 | 0.0142 | 78.255 | No |
| Tarng D., et al. 1998 | 0.794 (95% CI: 0.582; 1.005) | 0.000 | 4.0482 | 0.6023 | 6.989 | **Yes** |
| Sedighi O., et al. 2013 | 0.971 (95% CI: 0.583; 1.359) | 0.877 | -0.1665 | 0.0284 | 81.366 | No |
| Zhang K., et al. 2013 | 0.932 (95% CI: 0.564; 1.301) | 0.884 | 0.0362 | 0.0013 | 85.861 | No |
| Shahrbanoo et al. 2008 | 0.913 (95% CI: 0.519; 1.307) | 0.883 | 0.1349 | 0.0191 | 85.654 | No |
| Attallah N., et al. 2006 | 0.937 (95% CI: 0.530; 1.344) | 0.873 | 0.0085 | 0.00008 | 78.860 | No |
| Hajian S., et al. 2022 | 0.938 (95% CI: 0.542; 1.334) | 0.881 | 0.0069 | 0.00005 | 84.033 | No |
| Jalalzadeh M., et al. 2012 | 0.967 (95% CI: 0.586; 1.347) | 0.880 | -0.1466 | 0.0217 | 83.307 | No |
| Behairy M., et al. 2021 | 1.031 (95% CI: 0.666; 1.397) | 0.856 | -0.5175 | 0.2421 | 69.683 | No |
| El-Sharkawy M., et al. 2013 | 0.927 (95% CI: 0.521; 1.333) | 0.878 | 0.0632 | 0.0044 | 82.013 | No |
| Kang D., et al. 2012 | 0.933 (95% CI: 0.529; 1.337) | 0.878 | 0.0314 | 0.0011 | 82.290 | No |
| Keven K., et al. 2003 | 0.954 (95% CI: 0.563; 1.345) | 0.880 | -0.0794 | 0.0065 | 83.283 | No |

Supplementary Table 10. Subgroup analysis: TSAT

| **Variable** | **Subgroup** | **k** | **MD (95% CI)** | **I² (%)** | **p-value (Between Groups)** |
| --- | --- | --- | --- | --- | --- |
| **Risk of bias** | Low | 9 | 5.8779 (-0.0496 to 11.8054) | 94.2 | 0.1104 |
|  | High | 1 | 13.2700 (9.5029 to 17.0371) | - |  |
|  | Some concerns | 2 | 7.9205 (-8.0183 to 23.8593) | 97.4 |  |
| **Route of administration** | IV | 11 | 7.93 (3.06 to 12.80) | 97.0 | 0.0005 |
|  | Oral | 1 | –5.01 (–10.48 to 0.46) | — |  |
| **Study design** | RCT | 11 | 7.49 (2.34 to 12.65) | 97.0 | 0.0690 |
|  | Cross-over | 1 | –1.10 (–8.79 to 6.59) | — |  |
| **Follow-up** | 6 months | 3 | 6.94 (–4.40 to 18.28) | 96.8 | 0.7737 |
|  | 2 months | 3 | 10.93 (–0.68 to 22.55) | 98.5 |  |
|  | 3 months | 5 | 4.68 (–3.37 to 12.73) | 92.1 |  |
|  | 0.23 months | 1 | 4.10 (–2.47 to 10.67) | — |  |
| **Dose of Ascorbic Acid a month (mg)** | <2000 | 1 | 0.70 (0.12 to 1.27) | — | 0.1521 |
|  | 2000–5000 | 4 | 1.19 (0.50 to 1.89) | 92.7 |  |
|  | >5000 | 3 | 0.88 (0.46 to 1.30) | 0.0 |  |
|  | <5000* | 1 | 0.11 (–0.58 to 0.79) | — |  |
| **Frequency/week** | 3 first week | 1 | 0.70 (0.12 to 1.28) | — | 0.5732 |
|  | 3 | 7 | 1.00 (0.51 to 1.49) | 91.3 |  |
|  | 2 | 1 | 0.53 (–0.36 to 1.41) | — |  |
| **Center type** | Single-center | 9 | 6.24 (0.11 to 12.37) | 96.8 | 0.5723 |
|  | Multi-center | 3 | 9.07 (1.38 to 16.76) | 75.9 |  |
| **Year group** | After 2015 | 4 | 3.33 (–6.56 to 13.21) | 97.3 | 0.3434 |
|  | Before 2015 | 8 | 8.78 (3.34 to 14.22) | 92.5 |  |

Supplementary Table 11. Influence analysis: TSat levels

| **Author** | **Effect (95% CI: Lower; Upper)** | **I²** | **DFFITS** | **Cook's D** | **QE (del)** | **Is Influential** |
| --- | --- | --- | --- | --- | --- | --- |
| El Shinnawy H., et al. 2021 | 5.803 (95% CI: 0.881; 10.726) | 0.956 | 0.4394 | 0.1754 | 225.53 | No |
| Tarng D., et al. 1998 | 5.658 (95% CI: 0.917; 10.399) | 0.960 | 0.5186 | 0.2273 | 247.36 | No |
| Sedighi O., et al. 2013 | 7.427 (95% CI: 2.256; 12.597) | 0.970 | -0.2251 | 0.0517 | 333.37 | No |
| Shahrbanoo et al. 2008 | 6.368 (95% CI: 1.051; 11.685) | 0.966 | 0.1878 | 0.0377 | 296.72 | No |
| Tarng D., et al. 2004 | 7.076 (95% CI: 1.737; 12.415) | 0.970 | -0.0846 | 0.0077 | 332.02 | No |
| Attallah N., et al. 2006 | 7.210 (95% CI: 1.845; 12.575) | 0.969 | -0.1353 | 0.0199 | 322.23 | No |
| Hajian S., et al. 2022 | 7.494 (95% CI: 2.275; 12.712) | 0.970 | -0.2507 | 0.0646 | 333.40 | No |
| Behairy M., et al. 2021 | 7.931 (95% CI: 3.062; 12.800) | 0.970 | -0.4512 | 0.1833 | 329.98 | No |
| Bashardoust B., et al. 2017 | 7.536 (95% CI: 2.314; 12.758) | 0.939 | -0.2676 | 0.0734 | 164.01 | No |
| El-Sharkawy M., et al. 2013 | 6.242 (95% CI: 0.983; 11.500) | 0.965 | 0.2391 | 0.0597 | 286.73 | No |
| Kang D., et al. 2012 | 6.011 (95% CI: 0.926; 11.097) | 0.966 | 0.3396 | 0.1129 | 296.18 | No |
| Keven K., et al. 2003 | 7.494 (95% CI: 2.338; 12.650) | 0.970 | -0.2524 | 0.0646 | 333.31 | No |

Supplementary Table 12. Subgroup analysis: Ferritin

| **Variable** | **Subgroup** | **k** | **MD (95% CI)** | **I² (%)** | **p-value (Between Groups)** |
| --- | --- | --- | --- | --- | --- |
| **Risk of Bias** | Low | 7 | −24.63 (−94.72 to 45.46) | 64.04 | 0.1544 |
|  | High | 3 | −233.17 (−434.09 to −32.26) | 16.7 |  |
|  | Some concerns | 3 | −16.63 (−287.14 to 253.88) | 0.0 |  |
| **Route of Administration** | IV | 11 | −69.48 (−133.08 to −5.89) | 60.2 | 0.6355 |
|  | Oral | 2 | 1.31 (−284.43 to 287.05) | 61.3 |  |
| **Study Design** | RCT | 11 | −64.30 (−128.16 to −0.45) | 62.8 | 0.5746 |
|  | Cross-over | 2 | −96.09 (−186.88 to −5.29) | 0.0 |  |
| **Follow-up (months)** | 6.00 | 2 | 20.19 (−44.01 to 84.39) | 0.0 | 0.0418 |
|  | 2.00 | 3 | −144.04 (−259.95 to −28.14) | 35.6 |  |
|  | 3.00 | 7 | −73.11 (−117.17 to −29.05) | 58.6 |  |
|  | 0.23 | 1 | −31.00 (−209.68 to 147.68) | — |  |
| **Dose of Ascorbic Acid a month (mg)** | <2000 | 2 | 0.65 (0.12 to 1.18) | 0.0 | 0.1307 |
|  | 2000–5000 | 4 | 1.19 (0.50 to 1.89) | 92.7 |  |
|  | >5000 | 3 | 0.94 (0.46 to 1.41) | 0.0 |  |
|  | <5000 | 1 | 0.11 (−0.58 to 0.80) | — |  |
| **Frequency / Week** | 3 first week | 2 | 0.65 (0.12 to 1.18) | 0.0 | 0.6012 |
|  | 3 | 6 | 1.04 (0.49 to 1.59) | 92.3 |  |
|  | 2 | 1 | 0.53 (−0.36 to 1.42) | — |  |
|  | 7 | 1 | 3.20 (−3.58 to 9.98) | — |  |
| **Center Type** | Single-center | 6 | 0.99 (0.42 to 1.55) | 92.8 | 0.6685 |
|  | Multi-center | 4 | 0.83 (0.41 to 1.25) | 0.0 |  |
| **Year Group** | After 2015 | 3 | 0.55 (0.12 to 0.99) | 20.5 | 0.1193 |
|  | Pre-2015 | 7 | 1.10 (0.57 to 1.62) | 88.6 |  |

Supplementary Table 13. Influence analysis: Ferritin

| **Author** | **Effect (95% CI: Lower; Upper)** | **I²** | **DFFITS** | **Cook's D** | **QE (del)** | **Is Influential** |
| --- | --- | --- | --- | --- | --- | --- |
| El Shinnawy H., et al. 2021 | -77.64 (95% CI: -123.50; -31.79) | 0.523 | 0.589 | 0.225 | 23.06 | No |
| Tarng D., et al. 1998 | -52.06 (95% CI: -104.85; 0.73) | 0.554 | -0.531 | 0.236 | 24.69 | No |
| Sedighi O., et al. 2013 | -69.30 (95% CI: -128.82; -9.78) | 0.606 | 0.153 | 0.026 | 27.94 | No |
| Zhang K., et al. 2013 | -61.90 (95% CI: -123.85; 0.05) | 0.594 | -0.104 | 0.014 | 27.12 | No |
| Shahrbanoo et al. 2008 | -59.69 (95% CI: -113.32; -6.06) | 0.592 | -0.199 | 0.040 | 26.96 | No |
| Tarng D., et al. 2004 | -69.18 (95% CI: -127.73; -10.63) | 0.606 | 0.150 | 0.025 | 27.91 | No |
| Attallah N., et al. 2006 | -76.45 (95% CI: -134.50; -18.40) | 0.576 | 0.414 | 0.185 | 25.93 | No |
| Hajian S., et al. 2022 | -53.98 (95% CI: -100.51; -7.45) | 0.363 | -0.420 | 0.171 | 17.27 | No |
| Jalalzadeh M., et al. 2012 | -66.50 (95% CI: -119.60; -13.40) | 0.604 | 0.056 | 0.003 | 27.78 | No |
| Behairy M., et al. 2021 | -70.34 (95% CI: -123.29; -17.39) | 0.576 | 0.200 | 0.040 | 25.93 | No |
| Bashardoust B., et al. 2017 | -66.68 (95% CI: -119.25; -14.11) | 0.590 | 0.063 | 0.004 | 26.83 | No |
| El-Sharkawy M., et al. 2013 | -57.51 (95% CI: -107.74; -7.29) | 0.560 | -0.284 | 0.079 | 25.00 | No |
| Kang D., et al. 2012 | -71.70 (95% CI: -140.49; -2.91) | 0.607 | 0.192 | 0.063 | 27.98 | No |

Supplementary Table 14. Subgroup analysis: Iron levels.

| **Variable** | **Subgroup** | **k** | **MD (95% CI)** | **I² (%)** | **p-value (Between Groups)** |
| --- | --- | --- | --- | --- | --- |
| **Risk of bias** | Low | 7 | 10.9733 ( -2.2328 to 24.1795) | 86.7 | 0.6141 |
|  | High | 2 | -41.0610 (-155.2222 to 73.1002) | 94.9 |  |
|  | Some Concerns | 1 | 4.5100 ( -18.079 to 27.0997) | - |  |
| **Route of administration** | IV | 9 | 5.26 (–12.69 to 23.21) | 87.0 | 0.3295 |
|  | Oral | 1 | –6.84 (–23.26 to 9.58) | — |  |
| **Study design** | RCT | 9 | 10.99 (0.79 to 21.20) | 83.0 | <0.0001 |
|  | Cross-over | 1 | –102.20 (–153.15 to –51.25) | — |  |
| **Follow-up (months)** | 6 | 1 | 7.28 (–2.27 to 16.83) | — | 0.3636 |
|  | 2 | 3 | 18.47 (–0.86 to 37.80) | 80.3 |  |
|  | 3 | 6 | –6.32 (–36.26 to 23.62) | 87.0 |  |
| **Dose of Ascorbic Acid a month (mg)** | <2000 | 2 | 0.65 (0.12 to 1.18) | 0.0 | 0.1588 |
|  | 2000–5000 | 3 | 1.28 (0.34 to 2.21) | 92.1 |  |
|  | >5000 | 2 | 0.93 (0.45 to 1.40) | 0.0 |  |
|  | <5000 (single study) | 1 | 0.11 (–0.58 to 0.80) | — |  |
| **Frequency/week** | 3 first week | 2 | 0.65 (0.12 to 1.18) | 0.0 | 0.5530 |
|  | 3 | 5 | 1.06 (0.39 to 1.72) | 92.6 |  |
|  | 2 | 1 | 0.53 (–0.36 to 1.42) | — |  |
| **Center type** | Single-center | 5 | 0.99 (0.31 to 1.68) | 93.1 | 0.6773 |
|  | Multi-center | 3 | 0.82 (0.40 to 1.24) | 0.0 |  |
| **Year group** | After 2015 | 3 | 0.55 (0.12 to 0.99) | 20.5 | 0.1622 |
|  | Pre-2015 | 5 | 1.11 (0.47 to 1.75) | 89.9 |  |

Supplementary Table 15. Sensitivity analysis: Iron levels

| **Author** | **Effect (95% CI: Lower; Upper)** | **I²** | **DFFITS** | **Cook's D** | **QE (del)** | **Is Influential** |
| --- | --- | --- | --- | --- | --- | --- |
| El Shinnawy H., et al. 2021 | 3.18 (−15.74; 22.09) | 0.877 | 0.1502 | 0.0308 | 65.18 | no |
| Tarng D., et al. 1998 | 1.47 (−13.06; 16.00) | 0.821 | 0.4497 | 0.1536 | 44.61 | no |
| Sedighi O., et al. 2013 | 4.48 (−14.20; 23.15) | 0.832 | 0.0093 | 0.0001 | 47.59 | no |
| Shahrbanoo et al. 2008 | 3.68 (−14.75; 22.10) | 0.877 | 0.1003 | 0.0127 | 65.14 | no |
| Hajian S., et al. 2022 | 3.25 (−14.35; 20.86) | 0.877 | 0.1561 | 0.0276 | 65.24 | no |
| Jalalzadeh M., et al. 2012 | 11.00 (0.79; 21.20) | 0.830 | −0.9606 | 0.6638 | 46.98 | **yes** |
| Behairy M., et al. 2021 | 5.26 (−12.69; 23.21) | 0.870 | −0.0805 | 0.0078 | 61.64 | no |
| Bashardoust B., et al. 2017 | 4.60 (−13.20; 22.40) | 0.876 | −0.0044 | 0.00002 | 64.69 | no |
| El-Sharkawy M., et al. 2013 | 2.33 (−16.35; 21.01) | 0.873 | 0.2452 | 0.0800 | 62.91 | no |
| Kang D., et al. 2012 | 1.36 (−15.41; 18.12) | 0.850 | 0.3977 | 0.1650 | 53.41 | no |

Supplementary Table 16. Subgroup analysis: TIBC

| **Variable** | **Subgroup** | **k** | **MD (95% CI)** | **I² (%)** | **p-value (Between Groups)** |
| --- | --- | --- | --- | --- | --- |
| **Risk of bias** | Low | 5 | -33.5245 (-74.2893 to 7.2402) | 91.1 | 0.6300 |
|  | High | 3 | -13.2118 [ -23.5589; -2.8647] | 32.1 |  |
|  | Some concerns | 1 | −4.00 (−126.01 to 118.01) | — |  |
| **Route of administration** | IV | 8 | −30.69 (−54.29 to −7.08) | 81.0 | 0.0051 |
|  | Oral | 1 | 11.09 (−6.20 to 28.38) | — |  |
| **Study design** | RCT | 8 | −28.50 (−53.84 to −3.17) | 86.3 | 0.0783 |
|  | Cross-over | 1 | 5.00 (−22.37 to 32.37) | — |  |
| **Follow-up (months)** | 6 | 2 | −61.72 (−75.11 to −48.33) | 0.0 | <0.0001 |
|  | 3 | 5 | −3.69 (−26.28 to 18.91) | 50.4 |  |
|  | 2 | 2 | −13.64 (−25.83 to −1.45) | 0.0 |  |
| **Dose of Ascorbic Acid a month (mg)** | <2000 | 2 | 0.65 (0.12 to 1.18) | 0.0 | 0.1980 |
|  | 2000–5000 | 3 | 0.89 (0.57 to 1.21) | 0.0 |  |
|  | >5000 | 2 | 0.93 (0.45 to 1.40) | 0.0 |  |
|  | <5000 | 1 | 0.11 (−0.58 to 0.80) | — |  |
| **Frequency/week** | 3 first week | 2 | 0.65 (0.12 to 1.18) | 0.0 | 0.8646 |
|  | 2 | 2 | 0.53 (−0.36 to 1.42) | — |  |
|  | 3 | 5 | 0.82 (0.56 to 1.08) | 17.2 |  |
| **Center type** | Single-center | 6 | 0.75 (0.48 to 1.01) | 7.1 | 0.2952 |
|  | Multi-center | 3 | 0.82 (0.40 to 1.24) | 0.0 |  |
| **Year group** | After 2015 | 4 | 0.55 (0.12 to 0.99) | 20.5 | 0.6580 |
|  | Pre-2015 | 5 | 0.88 (0.60 to 1.15) | 0.0 |  |

Supplementary Table 17. Influence analysis: TIBC levels

| **Author** | **Effect (95% CI: Lower; Upper)** | **I^2** | **DFFITS** | **Cook's D** | **QE (del)** | **Is Influential** |
| --- | --- | --- | --- | --- | --- | --- |
| El Shinnawy H., et al. 2021 | -16.81 (95% CI: -40.04; 6.42) | 0.781 | -0.656963 | 0.345603 | 31.903 | No |
| Sedighi O., et al. 2013 | -22.04 (95% CI: -46.25; 2.17) | 0.872 | -0.151468 | 0.023076 | 54.750 | No |
| Attallah N., et al. 2006 | -17.41 (95% CI: -41.34; 6.52) | 0.833 | -0.576384 | 0.289413 | 41.803 | No |
| Hajian S., et al. 2022 | -26.15 (95% CI: -50.10; -2.21) | 0.871 | 0.190936 | 0.036473 | 54.285 | No |
| Jalalzadeh M., et al. 2012 | -28.50 (95% CI: -53.84; -3.17) | 0.863 | 0.386768 | 0.149638 | 51.047 | No |
| Behairy M., et al. 2021 | -30.69 (95% CI: -54.29; -7.08) | 0.810 | 0.624017 | 0.323496 | 36.811 | No |
| Bashardoust B., et al. 2017 | -24.47 (95% CI: -48.77; -0.17) | 0.874 | 0.050202 | 0.002539 | 55.422 | No |
| El-Sharkawy M., et al. 2013 | -25.57 (95% CI: -53.27; 2.12) | 0.865 | 0.130312 | 0.020332 | 51.752 | No |
| Kang D., et al. 2012 | -22.88 (95% CI: -50.31; 4.54) | 0.874 | -0.075395 | 0.006667 | 55.367 | No |

Supplementary Table 18. Subgroup analysis: EPO dosage Units/Week

| **Variable** | **Subgroup** | **k** | **MD (95% CI)** | **I² (%)** | **p-value (Between Groups)** |
| --- | --- | --- | --- | --- | --- |
| **Risk of bias** | High | 3 | −1360.64 (−2303.23 to −418.05) | 0.0 | 0.0069 |
|  | Low | 1 | 1420.50 (−363.27 to 3204.27) | — |  |
| **Route of administration** | Oral | 2 | 55.01 (−2541.24 to 2651.25) | 79.5 | 0.3678 |
|  | IV | 2 | −1350.50 (−2967.96 to 266.95) | 43.2 |  |
| **Study design** | Cross-over | 3 | −1360.64 (−2303.23 to −418.05) | 0.0 | 0.0069 |
|  | RCT | 1 | 1420.50 (−363.27 to 3204.27) | — |  |
| **Follow-up (months)** | 3 | 3 | −108.07 (−1684.22 to 1468.09) | 59.5 | 0.0787 |
|  | 6 | 1 | −2067.00 (−3578.28 to −555.72) | — |  |
| **Dose of Ascorbic Acid a month (mg)** | >5000 | 2 | 0.53 (−1.08 to 2.13) | 42.5 | 0.1386 |
|  | <2000 | 0 | — | — |  |
|  | <5000 | 1 | 1420.50 (−458.90 to 3299.90) | — |  |
| **Frequency/Week** | 7 | 1 | −1230.00 (−2766.99 to 306.99) | — | 0.7709 |
|  | 3 (first week) | 1 | −400.00 (−2344.85 to 1544.85) | — |  |
|  | 3 | 2 | −356.76 (−3773.82 to 3060.30) | 88.3 |  |
| **Center type** | Multi-center | 2 | −910.91 (−2116.79 to 294.97) | 0.0 | 0.7644 |
|  | Single center | 2 | −356.76 (−3773.82 to 3060.30) | 88.3 |  |
| **Year group** | Pre-2015 | 3 | −1360.64 (−2303.23 to −418.05) | 0.0 | 0.0069 |
|  | After 2015 | 1 | 1420.50 (−363.27 to 3204.27) | — |  |

Supplementary table 19. Influence analysis: EPO dosage Units/ Week

| **Author** | **Effect (95% CI: Lower; Upper)** | **I^2** | **DFFITS** | **Cook's D** | **QE (del)** | **Is Influential** |
| --- | --- | --- | --- | --- | --- | --- |
| Zhang K., et al. 2013 | -392.09 (95% CI: -2418.84; 1634.66) | 0.767 | -0.2632 | 0.0991 | 8.5812 | No |
| Jalalzadeh M., et al. 2012 | -673.21 (95% CI: -2691.65; 1345.22) | 0.776 | 0.0509 | 0.0036 | 8.9485 | No |
| Behairy M., et al. 2021 | -1360.64 (95% CI: -2303.23; -418.05) | 0.000 | 1.6398 | 0.9559 | 1.8042 | Yes |
| Keven K., et al. 2003 | -108.07 (95% CI: -1684.23; 1468.09) | 0.595 | -0.7621 | 0.4814 | 4.9346 | Yes |

Supplementary table 20. Assessment of the certainty of the evidence and summary of findings

| **Certainty assessment** | | | | | | | **№ of patients** | | **Effect** | **Certainty** |
| --- | --- | --- | --- | --- | --- | --- | --- | --- | --- | --- |
| **№ of studies** | **Study design** | **Risk of bias** | **Inconsistency** | **Indirectness** | **Imprecision** | **Other considerations** | **AA and Erythropoietin** | **Erythropoietin** | **Absolute (95% CI)** |  |
| **Hemoglobin levels (assessed with: Laboratory test)** | | | | | | | | | | |
| 12 | randomised trials | not serious | serious^a^ | not serious | not serious | none | 297 | 278 | MD **0.44 g/dL higher** (0.11 higher to 0.76 higher) | ⨁⨁⨁◯ Moderate^a^ |
| **Transferrin Saturation (assessed with: Laboratory test)** | | | | | | | | | | |
| 11 | randomised trials | not serious | serious^a^ | not serious | not serious | none | 246 | 233 | MD **7.21 % higher** (2.32 higher to 12.1 higher) | ⨁⨁⨁◯ Moderate^a^ |
| **Ferritin Levels (assessed with: Laboratory test)** | | | | | | | | | | |
| 13 | randomised trials | not serious | serious^a^ | not serious | serious^b^ | none | 309 | 300 | MD **90.26 ng/mL lower** (177.29 lower to 3.24 lower) | ⨁⨁◯◯ Low^a,b^ |
| **Iron Levels (assessed with: Laboratory test)** | | | | | | | | | | |
| 9 | randomised trials | not serious | serious^a^ | not serious | very serious^b,c^ | publication bias strongly suspected^d^ | 193 | 175 | MD **2.13 µg/dL higher** (18.31 lower to 22.57 higher) | ⨁◯◯◯ Very low^a,b,c,d^ |
| **Total Iron Binding Capacity (assessed with: Laboratory)** | | | | | | | | | | |
| 9 | randomised trials | not serious | serious^a^ | not serious | serious^b,c^ | publication bias strongly suspected^d^ | 216 | 183 | MD **22.54 µg/dL lower** (42.37 lower to 2.72 lower) | ⨁◯◯◯ Very low^a,b,c,d^ |
| **EPO dosage (assessed with: Laboratory test)** | | | | | | | | | | |
| 7 | randomised trials | not serious | serious^a^ | not serious | very serious^b,c^ | publication bias strongly suspected^d^ | 161 | 156 | MD **6.51 U/kg/month lower** (36.08 lower to 23.07 higher) | ⨁◯◯◯ Very low^a,b,c,d^ |

Supplementary table 20. **CI:** confidence interval; **MD:** mean difference **Explanations**: **a.** High I² values indicate substantial heterogeneity, **b.** Wide CI, **c.** Crosses 0, **d.** Funnel plot asymmetry observed.

Supplementary figure 1. Risk of bias assessment


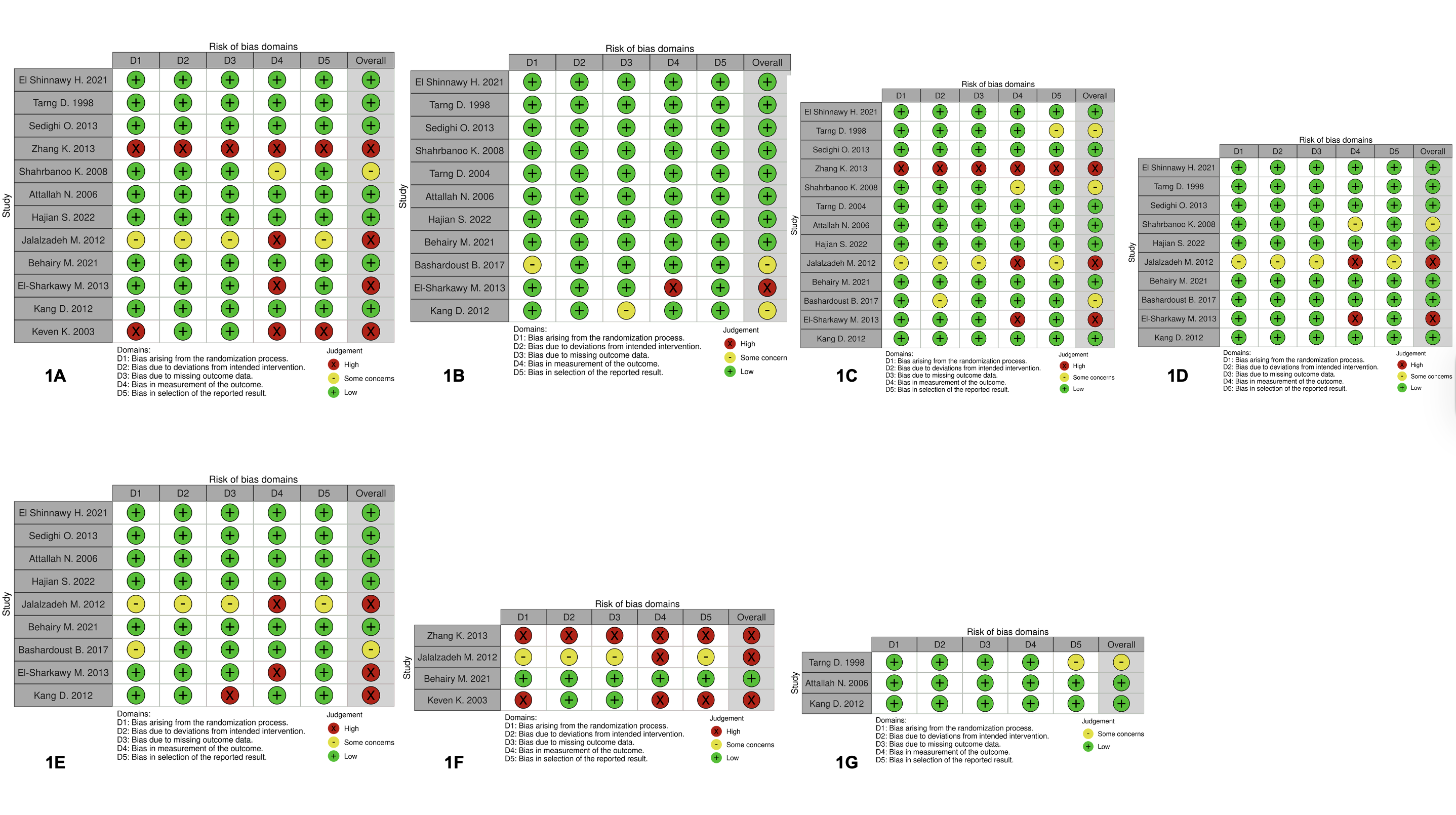


**Supplementary Figure 1.** Risk of bias assessment of randomized trials (RoB 2.0 tool). This figure represents a risk of bias summary table evaluating 7 outcomes across five bias domains (D1-D5). The domains included randomization, intervention deviations, missing data, outcome measurements, and reported result selection. The color-coded system-green for low risk, yellow for some concerns, and red for high risk-visually conveys the risk in each domain and overall, per study. Each outcome was individually assessed within its respective study. **Supplementary figure 1A.** Hemoglobin levels (g/dL); **Supplementary figure 1B.** TSAT (%); **Supplementary figure 1C.** Ferritin (ng/mL); **Supplementary figure 1D.** Iron level (µg/dL); **Supplementary figure 1E.** TIBC (µg/dL); **Supplementary figure 1F.** EPO dosage (Units/week); **Supplementary figure 1G.** EPO dosage (Units/kg/Week).

PRISMA 2020 checklist.

| **Topic** | **Item #** | **Checklist item** | **Location where item is reported** |
| --- | --- | --- | --- |
| **TITLE** | | |  |
| Title | 1 | Identify the report as a systematic review. | Page 1 |
| **ABSTRACT** | | |  |
| Abstract | 2 | See the PRISMA 2020 for Abstracts checklist. | N/A |
| **INTRODUCTION** | | |  |
| **Rationale** | 3 | Describe the rationale for the review in the context of existing knowledge. | Page 5 |
| **Objectives** | 4 | Provide an explicit statement of the objective(s) or question(s) the review addresses. | Page 5 |
| **METHODS** | | |  |
| **Eligibility criteria** | 5 | Specify the inclusion and exclusion criteria for the review and how studies were grouped for the syntheses. | Page 6 |
| **Information sources** | 6 | Specify all databases, registers, websites, organizations, reference lists and other sources searched or consulted to identify studies. Specify the date when each source was last searched or consulted. | Page 7 |
| **Search strategy** | 7 | Present the full search strategies for all databases, registers and websites, including any filters and limits used. | Page 7 |
| **Selection process** | 8 | Specify the methods used to decide whether a study met the inclusion criteria of the review, including how many reviewers screened each record and each report retrieved, whether they worked independently, and if applicable, details of automation tools used in the process. | Page 7 |
| **Data collection process** | 9 | Specify the methods used to collect data from reports, including how many reviewers collected data from each report, whether they worked independently, any processes for obtaining or confirming data from study investigators, and if applicable, details of automation tools used in the process. | Page 9 |
| **Data items** | 10a | List and define all outcomes for which data were sought. Specify whether all results that were compatible with each outcome domain in each study were sought (e.g. for all measures, time points, analyses), and if not, the methods used to decide which results to collect. | Page 8 |
|  | 10b | List and define all other variables for which data were sought (e.g. participant and intervention characteristics, funding sources). Describe any assumptions made about any missing or unclear information. | Page 7-8 |
| **Study risk of bias assessment** | 11 | Specify the methods used to assess risk of bias in the included studies, including details of the tool(s) used, how many reviewers assessed each study and whether they worked independently, and if applicable, details of automation tools used in the process. | Page 8 |
| **Effect measures** | 12 | Specify for each outcome the effect measure(s) (e.g. risk ratio, mean difference) used in the synthesis or presentation of results. | Page 9 |
| **Synthesis methods** | 13a | Describe the processes used to decide which studies were eligible for each synthesis (e.g. tabulating the study intervention characteristics and comparing against the planned groups for each synthesis (item #5)). | Page 7-8 |
|  | 13b | Describe any methods required to prepare the data for presentation or synthesis, such as handling of missing summary statistics, or data conversions. | Page 8 |
|  | 13c | Describe any methods used to tabulate or visually display results of individual studies and syntheses. | Page 7-8 |
|  | 13d | Describe any methods used to synthesize results and provide a rationale for the choice(s). If meta-analysis was performed, describe the model(s), method(s) to identify the presence and extent of statistical heterogeneity, and software package(s) used. | Page 9 |
|  | 13e | Describe any methods used to explore possible causes of heterogeneity among study results (e.g. subgroup analysis, meta-regression). | Page 9 |
|  | 13f | Describe any sensitivity analyses conducted to assess robustness of the synthesized results. | Page 9 |
| **Reporting bias assessment** | 14 | Describe any methods used to assess risk of bias due to missing results in a synthesis (arising from reporting biases). | Page 9 |
| **Certainty assessment** | 15 | Describe any methods used to assess certainty (or confidence) in the body of evidence for an outcome. | Page 9 |
| **RESULTS** | | |  |
| **Study selection** | 16a | Describe the results of the search and selection process, from the number of records identified in the search to the number of studies included in the review, ideally using a flow diagram. | Page 9-10 |
|  | 16b | Cite studies that might appear to meet the inclusion criteria, but which were excluded, and explain why they were excluded. | N/A |
| **Study characteristics** | 17 | Cite each included study and present its characteristics. | Page 9-10 |
| **Risk of bias in studies** | 18 | Present assessments of risk of bias for each included study. | Page 12 |
| **Results of individual studies** | 19 | For all outcomes, present, for each study: (a) summary statistics for each group (where appropriate) and (b) an effect estimate and its precision (e.g. confidence/credible interval), ideally using structured tables or plots. | Page 12-16 |
| **Results of syntheses** | 20a | For each synthesis, briefly summarise the characteristics and risk of bias among contributing studies. | Page 12-16 |
|  | 20b | Present results of all statistical syntheses conducted. If meta-analysis was done, present for each the summary estimate and its precision (e.g. confidence/credible interval) and measures of statistical heterogeneity. If comparing groups, describe the direction of the effect. | Page 12-16 |
|  | 20c | Present results of all investigations of possible causes of heterogeneity among study results. | Page 12-16 |
|  | 20d | Present results of all sensitivity analyses conducted to assess the robustness of the synthesized results. | Page 12-16 |
| **Reporting biases** | 21 | Present assessments of risk of bias due to missing results (arising from reporting biases) for each synthesis assessed. | Page 11-14 |
| **Certainty of evidence** | 22 | Present assessments of certainty (or confidence) in the body of evidence for each outcome assessed. | Page 12-16 |
| **DISCUSSION** | | |  |
| Discussion | 23a | Provide a general interpretation of the results in the context of other evidence. | Page 19-20 |
|  | 23b | Discuss any limitations of the evidence included in the review. | Page 21 |
|  | 23c | Discuss any limitations of the review processes used. | Page 21 |
|  | 23d | Discuss implications of the results for practice, policy, and future research. | Page 19-20 |
| **OTHER INFORMATION** | | |  |
| **Registration and protocol** | 24a | Provide registration information for the review, including register name and registration number, or state that the review was not registered. | Page 3 |
|  | 24b | Indicate where the review protocol can be accessed, or state that a protocol was not prepared. | N/A |
|  | 24c | Describe and explain any amendments to information provided at registration or in the protocol. | N/A |
| **Support** | 25 | Describe sources of financial or non-financial support for the review, and the role of the funders or sponsors in the review. | Page 17 |
| **Competing interests** | 26 | Declare any competing interests of review authors. | Page 17 |
| **Availability of data, code and other materials** | 27 | Report which of the following are publicly available and where they can be found: template data collection forms; data extracted from included studies; data used for all analyses; analytic code; any other materials used in the review. | N/A |

*From:*  Page MJ, McKenzie JE, Bossuyt PM, Boutron I, Hoffmann TC, Mulrow CD, et al. The PRISMA 2020 statement: an updated guideline for reporting systematic reviews. BMJ 2021;372:n71. doi: 10.1136/bmj.n71

PRISMA 2020 Abstract checklist.

| **Topic** | **Item #** | **Checklist item** | **Reported?** |
| --- | --- | --- | --- |
| **TITLE** | | |  |
| Title | 1 | Identify the report as a systematic review. | Yes |
| **BACKGROUND** | | |  |
| Objectives | 2 | Provide an explicit statement of the main objective(s) or question(s) the review addresses. | Yes |
| **METHODS** | | |  |
| Eligibility criteria | 3 | Specify the inclusion and exclusion criteria for the review. | N/A |
| Information sources | 4 | Specify the information sources (e.g. databases, registers) used to identify studies and the date when each was last searched. | Yes |
| Risk of bias | 5 | Specify the methods used to assess risk of bias in the included studies. | N/A |
| Synthesis of results | 6 | Specify the methods used to present and synthesise results. | Yes |
| **RESULTS** | | |  |
| Included studies | 7 | Give the total number of included studies and participants and summarise relevant characteristics of studies. | Yes |
| Synthesis of results | 8 | Present results for main outcomes, preferably indicating the number of included studies and participants for each. If meta-analysis was done, report the summary estimate and confidence/credible interval. If comparing groups, indicate the direction of the effect (i.e. which group is favoured). | Yes |
| **DISCUSSION** | | |  |
| Limitations of evidence | 9 | Provide a brief summary of the limitations of the evidence included in the review (e.g. study risk of bias, inconsistency and imprecision). | N/A |
| Interpretation | 10 | Provide a general interpretation of the results and important implications. | N/A |
| **OTHER** | | |  |
| Funding | 11 | Specify the primary source of funding for the review. | N/A |
| Registration | 12 | Provide the register name and registration number. | Yes |

*From:*  Page MJ, McKenzie JE, Bossuyt PM, Boutron I, Hoffmann TC, Mulrow CD, et al. The PRISMA 2020 statement: an updated guideline for reporting systematic reviews. BMJ 2021;372:n71. doi: 10.1136/bmj.n71. This work is licensed under CC BY 4.0. To view a copy of this license, visit <https://creativecommons.org/licenses/by/4.0/>
